# Diagnosis of Parkinson's disease by investigating the inhibitory effect of serum components on P450 inhibition assay

**DOI:** 10.1038/s41598-022-10528-x

**Published:** 2022-04-22

**Authors:** Kohei Ihara, Ami Oguro, Hiromasa Imaishi

**Affiliations:** 1 Division of Signal Responses, Biosignal Research Center, Kobe University, 1-1 Rokkodai, Nada, Kobe, 657-8501 Japan; 2 Program of Biomedical Science, Graduate School of Integrated Sciences for Life, Hiroshima University, Hiroshima, 739-8521 Japan

**Keywords:** Diagnostic markers, Prognostic markers

## Abstract

Parkinson’s disease (PD) is the second most common neurodegenerative disease, and diagnostic methods and biomarkers for patients without subjective motor symptoms have not yet been established. Previously, we developed a cytochrome P450 inhibition assay that detects alterations in metabolite levels associated with P450s caused by inflammation and exposure to endogenous or exogenous substances. However, it is unknown whether the P450 inhibition assay can be applied in PD diagnosis. Here, we determined whether the P450 inhibition assay can discriminate sera between patients with PD and healthy individuals. The results of the assay revealed that the P450 inhibition assay can discriminate PD with an area under the receiver operating characteristic curve (AUC) value of 0.814–0.914 in rats and an AUC value of 0.910 in humans. These findings demonstrate that the P450 inhibition assay can aid in the future development of liquid biopsy-based diagnostic methods for PD.

## Introduction

Parkinson’s disease (PD) is the second most common neurodegenerative disease and has been reported in over 6 million individuals worldwide as of 2016^1^. One of the pathological hallmarks of PD involves motor dysfunction caused by a decrease in the number of dopaminergic neurons in the substantia nigra pars compacta^2^. In recent years, microglia-mediated neuroinflammation has been shown to cause neurodegeneration of dopaminergic neurons^3,4^. Despite a large number of patients, effective diagnostic systems and biomarkers for patients without subjective motor symptoms have not yet been established. Consequently, the poor diagnostic options for PD delay the development of therapeutic approaches and medication for PD. Therefore, the development of efficient diagnostic systems and biomarkers is crucial for overcoming PD.

Cytochrome P450s (CYPs, P450s) are major drug-metabolizing enzymes that participate in phase I reactions as monooxygenases^5,6^. Substrates of P450s vary from endogenous to exogenous chemicals, and more than 3,000 substrates of P450s are known to exist^7^. The expression levels of P450s change under inflammatory conditions^8^. Under these conditions, proinflammatory cytokines such as interleukin-1β (IL-1β), interleukin-6 (IL-6), and tumor necrosis factor α (TNFα) regulate the expression of P450s via certain nuclear receptors or transcription factors such as nuclear factor-κB (NF-κB)^8^. In inflammatory diseases such as cancer^9^, cardiovascular disease^10^, diabetes^11,12^ and ulcerative colitis^13,14^, the expression of P450s differs from that in a disease-free state. Moreover, these changes in P450s vary among diseases^9,10^.

Considering the aforementioned features of P450s, we hypothesized that changes in the expression patterns of P450s affect the quantity and quality of metabolites associated with P450s in blood. Studying these changes may enable us to understand the disease states of donors. To detect changes in metabolites related to P450s, we previously developed a P450 inhibition assay^15^. In this assay, recombinant P450s expressed on the cell membrane of *Escherichia coli* are mixed with serum and the fluorescent substrate Vivid®^16–18^. Certain serum metabolites inhibit the P450-mediated oxidation of the fluorescent substrate. Changes in the inhibition rate of the Vivid® decomposition reaction reflect the quantity and quality of the serum metabolites associated with P450s. We performed this assay to successfully discriminate sera among ulcerative colitis model mice^15^, and type 1 and 2 diabetes model mice^19^. These results indicate that the P450 inhibition assay can be applied to other diseases that involve changes in P450 expression.

Neuroinflammation is one of the pathological hallmarks of PD. It is well known that altered levels of NF-κB are involved in the degeneration of dopaminergic neurons^20^. Moreover, the levels of proinflammatory cytokines such as IL-1β, IL-6, and TNFα are increased in the blood and cerebrospinal fluid (CSF)^21–23^. Therefore, the expression levels of P450s in patients with PD are likely to vary from those in healthy individuals. It has been reported that CYP2E1 is upregulated in the brains of patients with PD^24^. Furthermore, it has been reported that damage to the dopaminergic neurons in the brains alters the expression levels of liver P450s in rats^25^. P450s are also involved in the biosynthesis and catabolism of neurotransmitters, neurotoxins, and neuroprotective hormones, which are involved in neurodegenerative disease progression^26^. Certain polymorphisms in genes encoding CYP1A1 and CYP2D6, which metabolize endogenous and exogenous toxins, increase the risk of PD^27,28^. Therefore, the pathology of PD is related to the function of P450s.

Based on these facts, we hypothesized that the P450 inhibition assay can discriminate sera between patients with PD and healthy individuals. To clarify whether symptoms of PD affect the results of the P450 inhibition assay, we established rotenone-injected PD model rats and evaluated their sera via P450 inhibition assays in this study. In addition, to clarify whether this method is applicable to the diagnosis of PD in humans, sera from patients with PD were also evaluated via P450 inhibition assays. Based on these results, we suggest that the P450 inhibition assay is potentially applicable to the diagnosis of patients with PD.

## Results

### Development of rotenone-induced PD model rats

**Figure 1:**
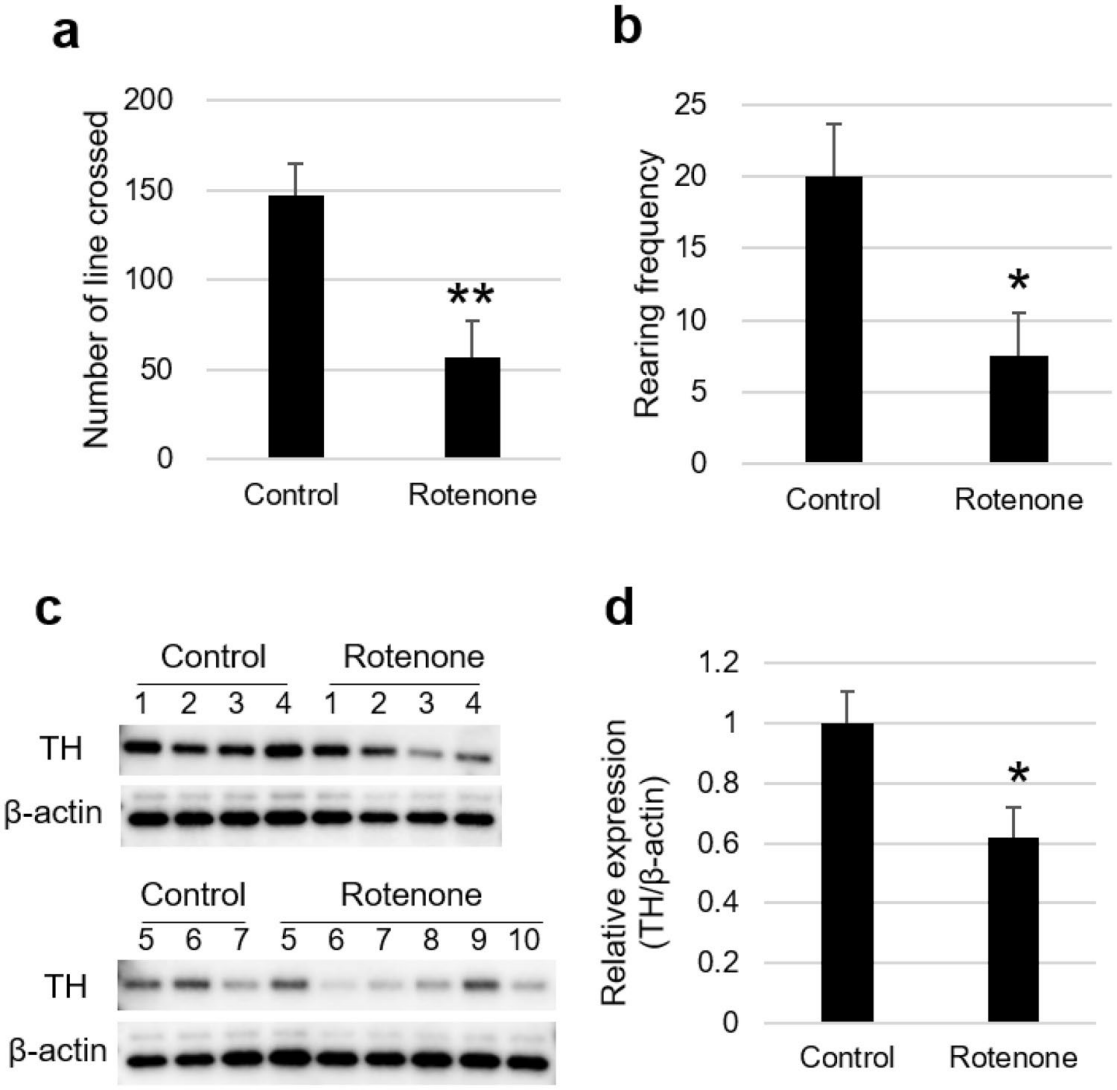
Pathological analysis of control rats (n = 7) and rotenone-administered Parkinson’s disease model rats (n = 10). (**a**) Number of lines crossed and (**b**) frequency of rearing in an open field test. (**c**) Expression levels of TH and b-actin in the rat striatum were analyzed via immunoblotting analyses. (**d**) Relative expression levels of TH were evaluated via immunoblotting analysis. Values are presented as the mean ± standard error of the mean. Significant differences were evaluated via Welch’s t-test (**P < 0.01, *P < 0.05). TH, tyrosine hydroxylase.

**Figure 2:**
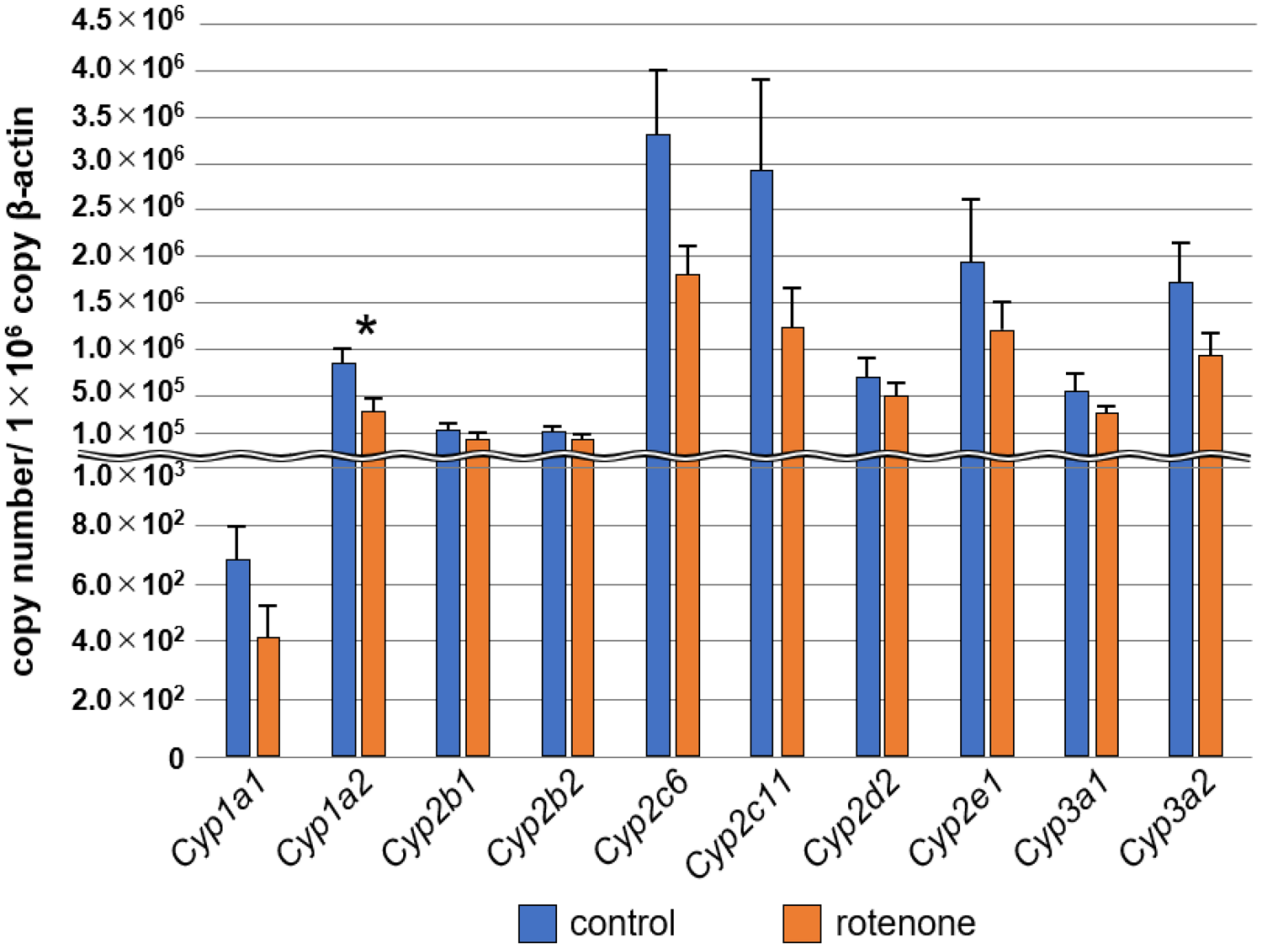
Transcriptional analysis of the P450 genes in rotenone-administered rats and a control rat. Total RNA was isolated from livers recovered from control rats (n = 7) and rotenone-administered rats (n = 10). Expression levels of each P450 were evaluated via quantitative polymerase chain reaction. Copy numbers of each P450 per 10^6^ b-actin copies are presented as the mean ± standard error of the mean. Significant differences were evaluated via Welch’s t-test (*P < 0.05).

To evaluate the applicability of the P450 inhibition assay, we established rotenone-induced PD model rats^29–31^ and evaluated their sera using the assay. After five days of serial administration of rotenone (3 mg/kg) to the rats, the motor function was evaluated using an open-field test. The control group that was not administered rotenone showed a locomotor activity of 146.9 ± 17.7 lines crossed (Fig. 1a). In contrast, the rotenone-administered group showed a considerably lower locomotor activity of 57.3 ± 19.8 lines crossed (Fig. 1a). The rearing frequency of the rotenone-administered group (7.6 ± 2.9) was significantly lower than that of the control group (20.0 ± 3.7) (Fig. 1b). To determine whether rotenone administration induced neurodegeneration in the rat brains, we evaluated the expression levels of tyrosine hydroxylase (TH) in the striatum via immunoblotting analysis. The TH expression level in the rotenone-administered group (0.62 ± 0.10) was significantly lower than that of the control group (1.00 ± 0.10) (Fig. 1c, d, and Supplementary Fig. S1). To determine whether the expression levels of P450s change in the liver of the PD model rats, reverse transcription-quantitative polymerase chain reaction (RT-qPCR) was performed. The expression levels of 10 rat P450s (*Cyp1a1*, *Cyp1a2*, *Cyp2b1*, *Cyp2b2*, *Cyp2c6*, *Cyp2c11*, *Cyp2d2*, *Cyp2e1*, *Cyp3a1*, and *Cyp3a2*) were 28.5–60.3% lower than those of the control group, and significant differences were observed in *Cyp1a2* expression levels (Fig. 2).

### P450 inhibition assays using sera from PD model rats

**Figure 3:**
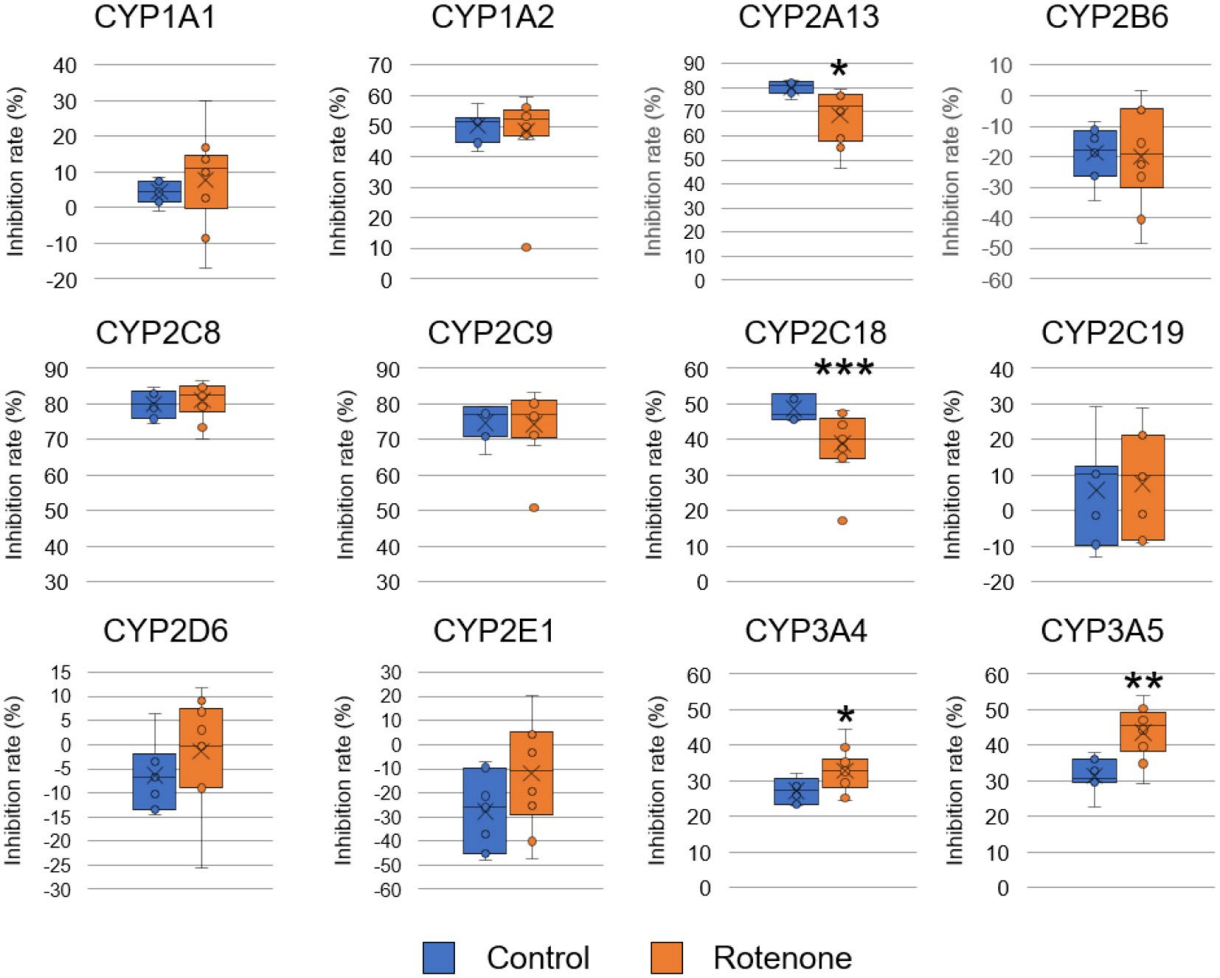
P450 inhibition assay using serum samples of Parkinson’s disease model rats. Inhibition rate of reactions in the sera recovered from control rats (n = 7) and rotenone-administered rats (n = 10) toward 12 types of P450s and fluorescent substrate reactions are presented. Values are presented in a box plot. The black solid bars within the box plot represent the median abundance, and the cross mark represents mean abundance. Significant differences were evaluated via Welch’s t-test (***P < 0.001, **P < 0.01, *P < 0.05).

**Figure 4:**
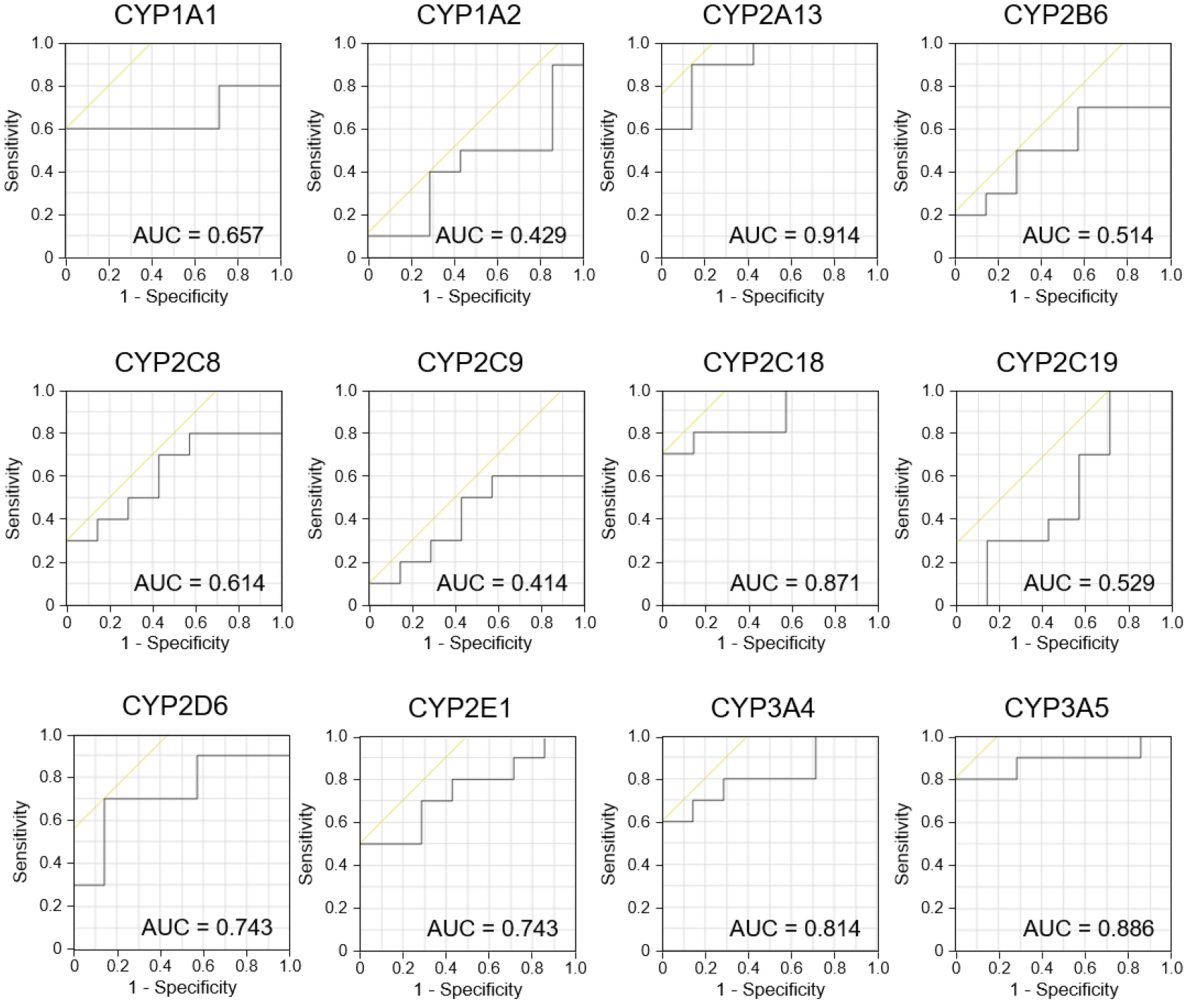
Receiver operating characteristic analysis of the P450 inhibition assay using Parkinson’s disease model rats. Values of the area under the curve are presented in each graph.

To evaluate whether the P450 inhibition assay can discriminate sera between PD model rats and control rats, we performed a P450 inhibition assay using sera recovered from rats. The inhibition rates of Vivid® decomposition reactions in the sera were determined using 12 human P450s (CYP1A1, CYP1A2, CYP2A13, CYP2B6, CYP2C8, CYP2C9, CYP2C18, CYP2C19, CYP2D6, CYP2E1, CYP3A4, and CYP3A5). First, to examine the effect of the remaining amount of rotenone in the sera, we quantitated the rotenone using high-performance liquid chromatography (HPLC) (Supplementary Fig. S2). HPLC results demonstrated that the concentrations of rotenone recovered from the sera of 10 rotenone-administrated rats were under the detection limit (< 0.05 μg/mL). We also demonstrated that this concentration (0.05 μg/mL) did not affect the P450 inhibition assay (Supplementary Fig. S3). These results indicate that the P450 inhibition assay was not affected by the amount of rotenone remaining in the sera. Next, we subjected the sera recovered from control and rotenone-administrated rats to the P450 inhibition assay. Significant differences in inhibition rates were observed for four P450s (Fig. 3). The inhibition rate of reactions in sera recovered from rotenone-administered groups for CYP2A13 and CYP2C18 was significantly lower than that in the control. In contrast, the inhibition rate of reactions in sera recovered from rotenone-administered groups for CYP3A4 and CYP3A5 was higher than that in the control. The other P450s (CYP1A1, CYP1A2, CYP2B6, CYP2C8, CYP2C9, CYP2C19, and CYP2E1) did not show any significant differences in reaction rates. Receiver operating characteristic (ROC) analyses were performed to evaluate the diagnostic performance of the assay (Fig. 4 and Supplementary Table S1). In ROC analysis, a graph is constructed to plot how the sensitivity (true positive rate) and specificity (true negative rate) of a test change depending on the test’s threshold value (inhibition rate)^32^. ROC analysis is one of the most commonly used methods in evaluating the accuracy of clinical tests. Certain P450s that did not show significant differences in the inhibition rate of reactions presented low or moderate area under the curve (AUC) values (0.414 to 0.743). CYP2A13, CYP2C18, CYP3A4, and CYP3A5, which showed significant differences, presented high AUC values of 0.914, 0.871, 0.814, and 0.866, respectively.

### Correlation between motor dysfunction and inhibition rate of reaction

**Figure 5:**
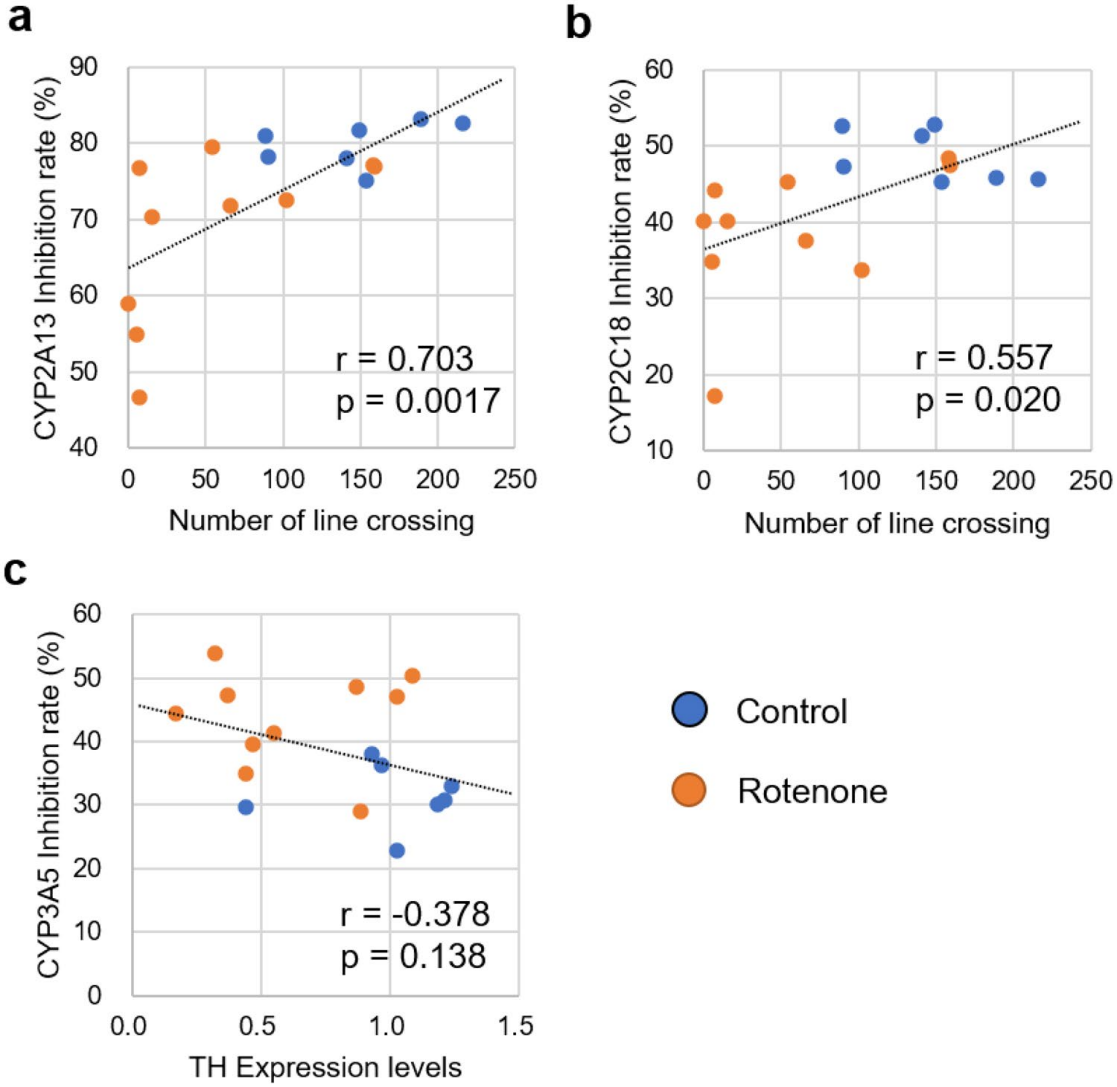
Correlation between the results of CYP inhibition and motor dysfunction or TH expression. (**a**) Scatter plot of the inhibition rate of CYP2A13 and number of lines crossed. (**b**) Scatter plot of the inhibition rate of CYP2C18 and number of lines crossed. (**c**) Scatter plot of the inhibition rates of CYP3A5 and TH expression levels. Pearson product-moment correlation coefficient (r) and the result of the test showing no correlation (p) are presented in each graph. CYP, cytochrome P450; TH, tyrosine hydroxylase.

The correlation between motor dysfunction and inhibition rate in the P450 inhibition assay was evaluated via Pearson's correlation coefficient analyses (Fig. 5). The number of lines crossed in the open field test and the inhibition rate associated with CYP2A13 were positively correlated (r = 0.703 and *P* = 0.0017) (Fig. 5a). The number of lines crossed and the inhibition rate associated with CYP2C18 also showed a positive correlation coefficient (r = 0.557, *P* = 0.020) (Fig. 5b). The TH expression level and inhibition rate of CYP3A5 showed a weak negative correlation; however, it was not significant (r =  − 0.378 and *P* = 0.138).

### P450 inhibition assay using clinical samples

**Figure 6:**
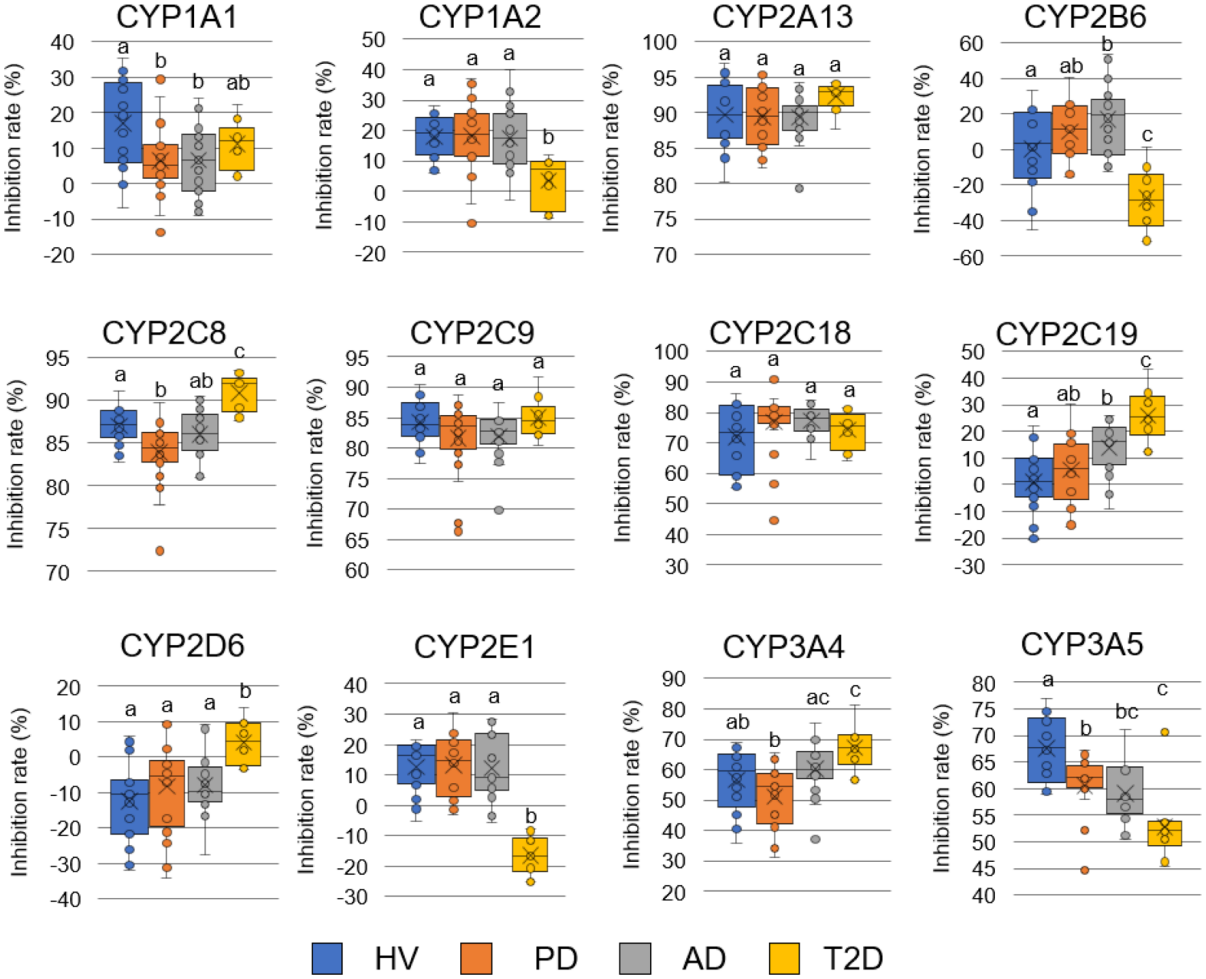
P450 inhibition assays using human clinical samples. Inhibitory rate of reactions in the sera recovered from HVs (n = 20), patients with PD (n = 20), patients with AD (n = 19), and patients with T2D (n = 10) toward 12 types of P450s and fluorescent substrate reactions are presented in a box plot. The black solid bars within the box plot represent the median abundance, and the Cross mark represents the mean abundance. Significant differences were evaluated via Tukey–Kramer test (*P* < 0.05). Means having the same letter above the graphs are not significantly different from each other. P450, cytochrome P450; HV, healthy volunteer; PD, Parkinson’s disease; AD, Alzheimer’s disease; T2D, type2 diabetes.

**Figure 7:**
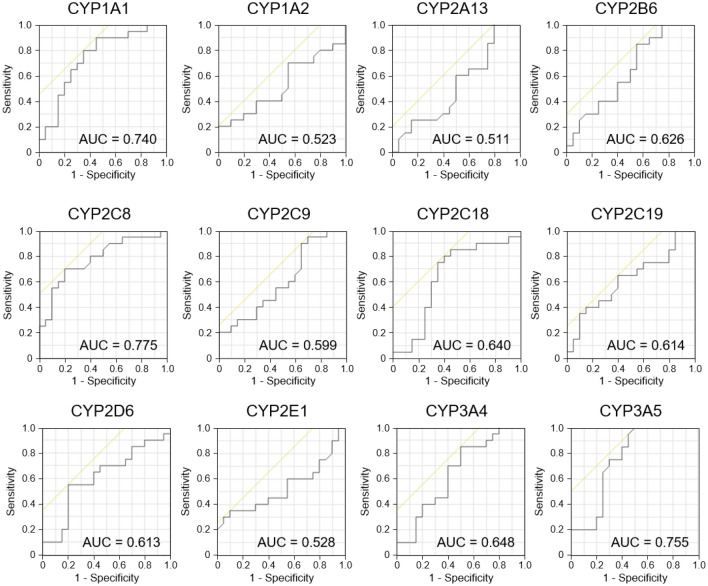
Receiver operating characteristic analysis of the P450 inhibition assay in patients with PD and healthy individuals. Values of the area under the curve are presented in each graph.

Human clinical samples were evaluated via the assay to determine whether the P450 inhibition assay can discriminate sera between patients with PD and healthy individuals. Sera from patients with PD (n = 20) and healthy volunteers (n = 20) were subjected to the P450 inhibition assay, and the inhibition rates of reactions associated with 12 human P450s (CYP1A1, CYP1A2, CYP2A13, CYP2B6, CYP2C8, CYP2C9, CYP2C18, CYP2C19, CYP2D6, CYP2E1, CYP3A4, and CYP3A5) were determined. We observed significant differences in inhibition rates for three P450s (Fig. 6). The inhibition rate associated with CYP1A1 was significantly lower than that of the control (6.4% and 17.0%, respectively). The inhibition rate associated with CYP2C8 was also lower than that of the control (83.9% and 87.0%, respectively). The inhibition rate associated with CYP3A5 was lower than that of the control (60.5% and 67.6%, respectively). In contrast, the inhibition rates associated with other P450s (CYP1A2, CYP2A13, CYP2B6, CYP2C9, CYP2C18, CYP2C19, CYP2E1, and CYP3A4) were not significantly different. To evaluate whether the P450 inhibition assay can be used in diagnosing PD, we subjected the sera from patients with Alzheimer’s disease (AD) and type 2 diabetes (T2D) to the assay. We observed significant differences in the inhibition rates of four P450s between healthy individuals and patients with AD (Fig. 6). The inhibition rates of CYP2B6 and CYP 2C19 were significantly higher than those of the healthy group while those of CYP 1A1 and CYP3A5 were significantly lower than those of the healthy group. Furthermore, significant differences were observed in the inhibition rates of CYP3A4 between patients with PD and AD (Fig. 6). These results indicate that the P450 inhibition assay can distinguish PD from AD, both of which are neurodegenerative diseases occurring in the brain. Moreover, this distinguishing ability of the P450 inhibition assay is also effective with regard to other inflammation-associated diseases such as T2D. To this extent, we observed significant differences in the inhibition rates of eight P450s between healthy individuals and patients with T2D (Fig. 6). The inhibition rates of CYP2C8, CYP2C19, CYP2D6, and CYP 3A4 were significantly higher than those of the healthy group, while those of CYP 1A2, CYP2B6, CYP2E1, and CYP3A5 were significantly lower than those of the healthy group. ROC analyses were performed to evaluate the diagnostic performance of the assay (Fig. 7 and Supplementary Table S2). The P450s that did not show significant differences in the inhibition rates presented AUC values of 0.511 to 0.648. In contrast, CYP1A1, CYP2C8, and CYP3A5, which showed significant differences, presented AUC values of 0.740, 0.775, and 0.755, respectively.

### Correlation between motor dysfunction (Hoehn and Yahr scale) and inhibition rate

**Figure 8:**
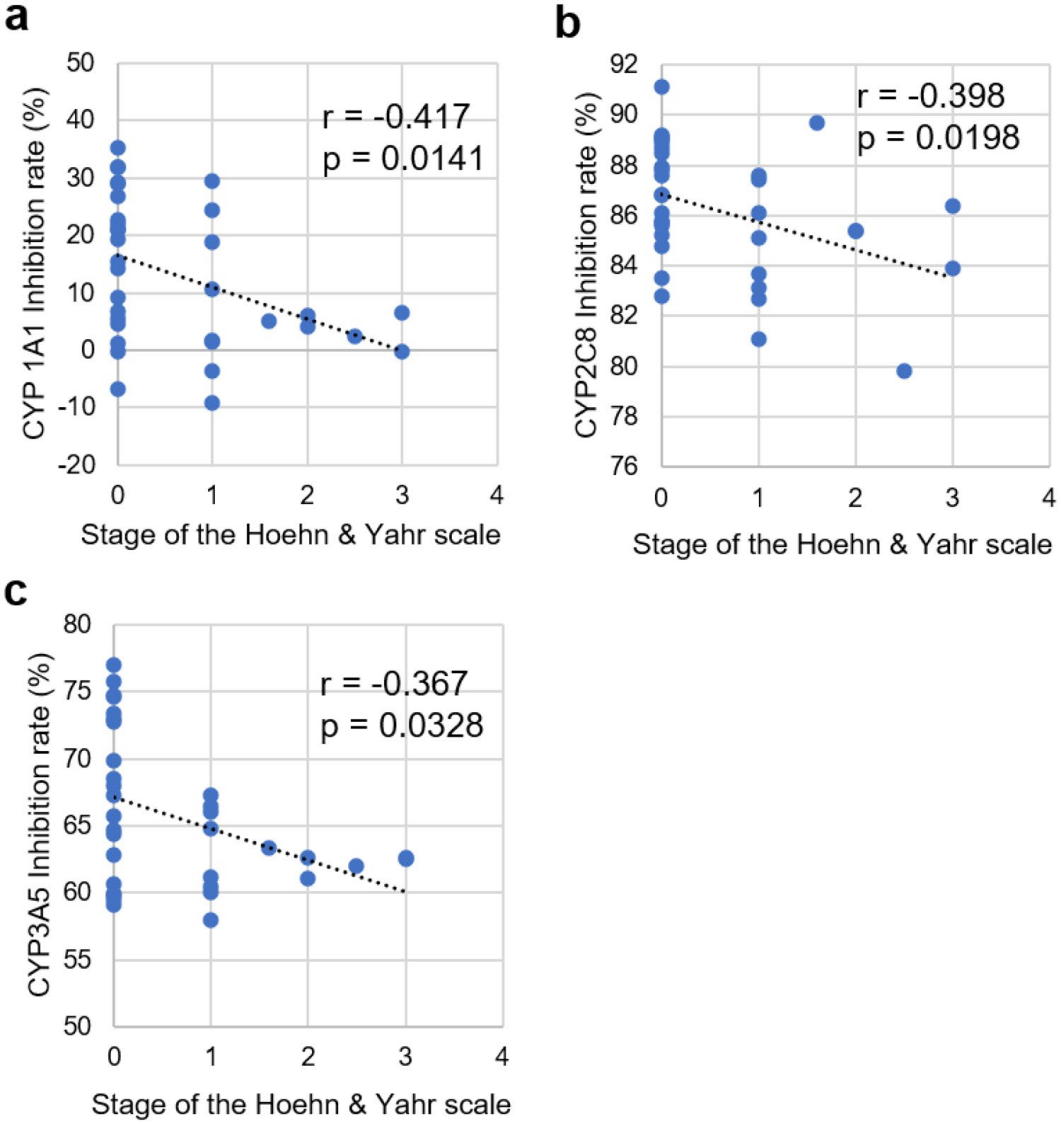
Correlation of inhibition rate of reactions involving CYP1A1, CYP2C8, and CYP3A5 and the stage of the Hoehn and Yahr scale. (**a**) Scatter plot of the inhibition rate of the reaction for CYP1A1 and the stage of the Hoehn and Yahr scale. (**b**) Scatter plot of the inhibition rate of the reaction for CYP2C8 and the stage of the Hoehn and Yahr scale. (**c**) Scatter plot of the inhibition rate of the reaction for CYP3A5 and the stage of the Hoehn and Yahr scale. Spearman's rank correlation coefficient (r) and the result of the test showing no correlation (p) are presented in each graph.

The correlation between the stage of the Hoehn and Yahr scale and the inhibition rate in the P450 inhibition assay was evaluated using Spearman’s rank correlation coefficient analysis (Fig. 8). The Hoehn and Yahr scale is an index comprised of 5 stages that characterize the degree of progression of symptoms associated with Parkinson's disease^33^. Stage 0 indicates no signs of disease, while stage 5 indicates bed or wheelchair confinement unless the patient was aided. The correlation between HY score and inhibition rate associated with CYP1A1, CYP2C8, and CYP3A5 indicated a negative correlation (r =  − 0.417, − 0.398, and − 0.367, respectively).

### Combination of multiple inhibition rates

**Figure 9:**
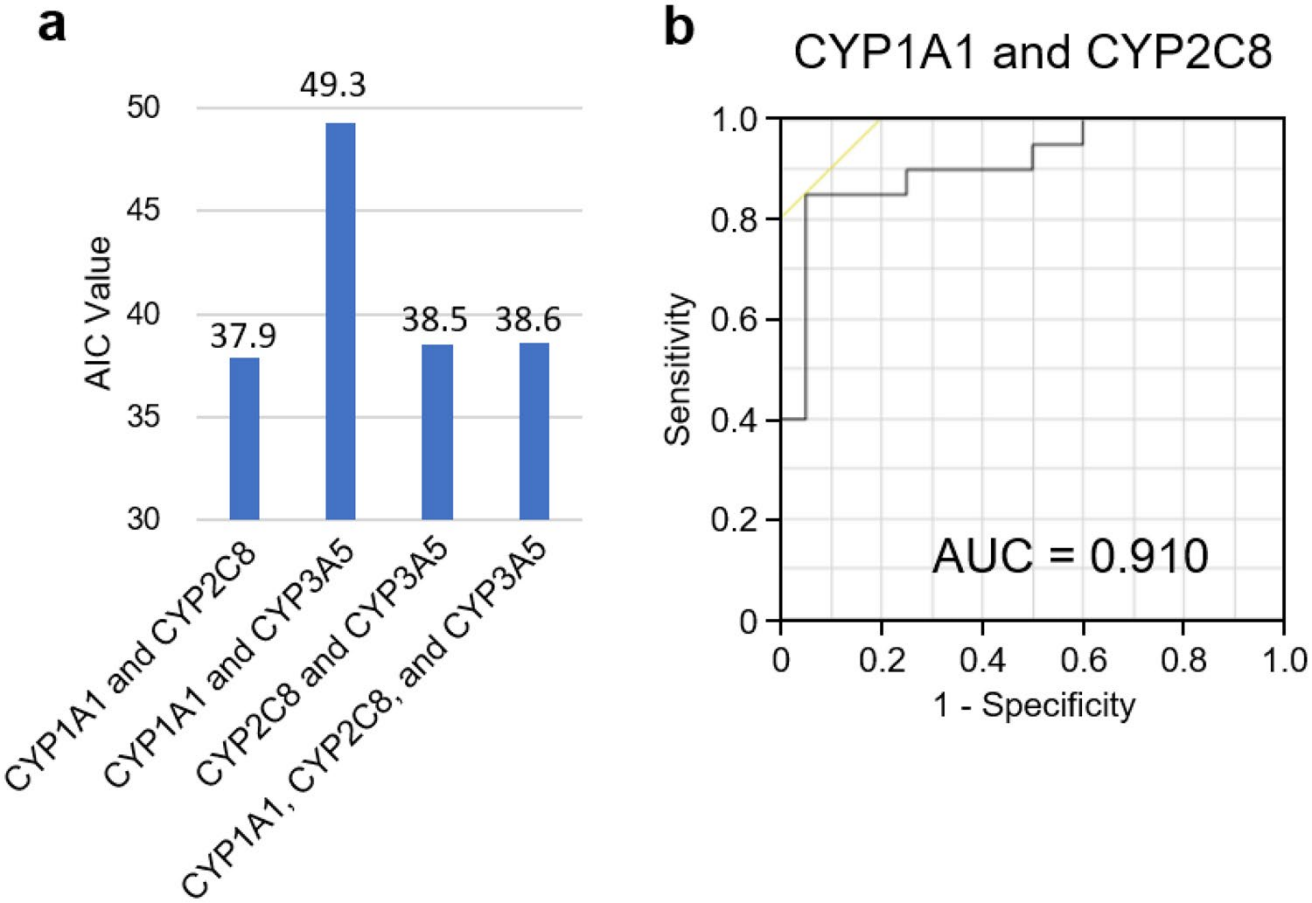
Evaluation of diagnosis efficiency based on the combination of CYP1A1, CYP2C8, and CYP3A5. (**a**) AIC of the logistic regression models was determined. AIC values for each combination of the P450s are presented in the graph. (**b**) Receiver operating characteristic analysis of a regression model which combined CYP1A1 and CYP2C8 as the best-fit model. Values of the area under the curve are presented in the graph. AIC, Akaike’s information criterion.

To evaluate the diagnostic efficiency of the P450 inhibition assay, logistic regression models were constructed using various combinations of three P450s, CYP1A1, CYP2C8, and CYP3A5, which demonstrated significant differences in inhibition rates (Fig. 9 and Supplementary Table S7). The Akaike's information criterion (AIC) values of the four regression models are shown in Fig. 9a. The combination of CYP1A1 and CYP2C8 was associated with the lowest AIC value (37.9), indicating that this model was the best-fit model for discriminating PD. To evaluate the diagnostic performance of this model, ROC analyses were performed using a combination of CYP1A1 and CYP2C8; the model showed an AUC value of 0.910 (Fig. 9b and Supplementary Table S3).

## Discussion

PD is the second most common neurodegenerative disease. However, diagnostic systems and biomarkers for patients without subjective motor symptoms have not yet been established. In our previous study, we developed a P450 inhibition assay to detect changes in P450-associated serum metabolites^15^. We used this assay to successfully discriminate between sera obtained from ulcerative colitis model mice^15^, and type 1 and 2 diabetes model mice^19^. This assay is based on the phenomenon that several diseases such as cancer^9^, cardiovascular disease^10^, diabetes^11,12^, and ulcerative colitis^13,14^ are associated with altered expression levels of P450s in patients. Moreover, changes in the expression levels of P450s vary among various diseases^9,10^. Inflammation alters the expression levels of P450s because proinflammatory cytokines, such as IL-1β, IL-6, and TNFα, regulate P450 expression via certain nuclear receptors or transcription factors such as NF-κB^8^. PD is known to induce inflammation^21–23^, which can alter the expression levels of P450s in patients with PD. It has been reported that PD and neurodegeneration of the dopaminergic neurons in the brains are associated with altered expression levels of P450s in the brain and liver^24,25^. Therefore, we hypothesized that the P450 inhibition assay can discriminate between sera from patients with PD and healthy individuals. In this study, we developed a PD animal model and performed a case–control study. The results of these experiments revealed that the P450 inhibition assay is potentially applicable for the diagnosis of patients with PD.

To evaluate whether the P450 inhibition assay can be used for PD diagnosis, we developed rotenone-administered PD model rats and evaluated the sera from these rats using the assay. The results of the assay showed that the inhibition rates of the reaction associated with four P450s, CYP2A13, CYP2C18, CYP3A4, and CYP3A5, were significantly altered (Fig. 3), and the AUC values ranged from 0.814 to 0.914 in the ROC analysis (Fig. 4 and Supplementary Table S1). Moreover, the inhibition rate of the reaction associated with CYP2A13 and CYP2A18 was correlated with motor dysfunction (Fig. 5), indicating that alterations in the inhibition rate reflect the progression of the disease symptoms. Therefore, we concluded that the P450 inhibition assay could discriminate between PD model rats and control rats. We evaluated the expression levels of liver P450s in rotenone-administered rats and found that the expression levels of 10 types of P450s (*Cyp1a1*, *Cyp1a2*, *Cyp2b1*, *Cyp2b2*, *Cyp2c6*, *Cyp2c11*, *Cyp2d2*, *Cyp2e1*, *Cyp3a1*, and *Cyp3a2*) were 28.5–60.3% lower than those in the control group. P450s are downregulated by inflammatory mediators such as IL-1β, IL-6, and TNFα via certain nuclear receptors or transcription factors such as NF-κB^8^. It is well known that rotenone administration causes inflammation^34^. Therefore, the expression levels of P450s in rotenone-administered rats were lower than those in the control rats which altered the inhibition rate in the P450 inhibition assay.

We successfully demonstrated that the P450 inhibition assay could discriminate PD model rats from normal rats, indicating that P450 alteration occurs as a result of the progression of the disease symptoms. In this study, the genetic background, sex, and breeding environment of the rats were identical. However, the expression levels of P450s are affected by various factors, such as genetic background, sex, and food intake^5,35^. Therefore, the applicability of this assay in human diagnosis should be further tested. We performed a P450 inhibition assay using sera recovered from patients with PD and healthy volunteers. The results of the assay showed that the inhibition rates for three P450s, CYP1A1, CYP2C8, and CYP3A5, were significantly altered (Fig. 6), and the AUC values ranged from 0.740 to 0.775 in the ROC analysis (Fig. 7 and Supplementary Table S2). We constructed a logistic regression model using CYP1A1 and CYP2C8 which was found to represent the best-fit model for discriminating PD. In this model, the AUC value was 0.910 (Fig. 9 and Supplementary Table S3), indicating that the P450 inhibition assay may be applicable for PD diagnosis in humans. Moreover, the inhibition rates associated with CYP1A1, CYP2C8, and CYP3A5 were correlated with the stage of the Hoehn and Yahr scale (Fig. 8). This result was similar to that observed using rats, which is correlated with motor dysfunction (Fig. 5). However, the inhibition rates determined using rat and human sera were completely different (Figs. 3, 6). According to the “Cytochrome P450 Homepage” (https://drnelson.uthsc.edu/)^36^, 57 species of P450s have been identified in humans; however, 89 species of P450s have been identified in rats. It is well known that differences in P450s between species result in varying metabolism of many substances^37^. Therefore, differences in the variation of P450s between humans and rats could be attributed to differences in the inhibition rates in the P450 inhibition assay.

To assess the applicability of the P450 inhibition assay in PD diagnosis, it was necessary to determine if this assay has the ability to distinguish other neurodegenerative diseases, such as AD, and inflammatory-associated diseases, such as T2D. Thus, we performed the P450 inhibition assay using the sera of patients with AD and T2D. The results demonstrated that the P450 inhibition assay can distinguish samples from patients with PD as well as other diseases (Fig. 6). These results indicate that the P450 inhibition assay can detect PD-specific changes that differentiate it from other neurodegenerative and inflammatory diseases. Moreover, these results indicate that the P450 inhibition assay could potentially be used in the diagnosis of AD and T2D.

The inhibition rates of reactions in sera from patients with PD and healthy volunteers were altered. We hypothesized that these alterations could be caused by changes in the expression levels of P450s in patients with PD. P450 expression is regulated by proinflammatory cytokines such as IL-1β, IL-6, and TNFα, which increase in level in the blood and CSF in patients with PD^21–23^. Furthermore, dysfunction in the dopaminergic system alters the expression levels of liver P450s in rats^25^. Furthermore, PD progression leads to the degeneration of the noradrenergic systems, which affects the neuroendocrine regulation of liver cytochrome P450^38^. These facts indicate that the expression levels of P450s in patients with PD may be different than those in healthy individuals. These alterations in P450 expression may affect the quantity and quality of related metabolites or inhibitors, such as neurotransmitters including tyrosine and dopamine metabolite 3,4-dihydroxyphenylacetic acid^39,40^ and neuroprotection substances such as caffeine^41^ and tocopherol^42,43^. The quantity of these substances is altered in the sera or CSF of patients with PD^44–46^. Thus, the P450 inhibition assay may reflect these differences and successfully discriminate between PD and healthy controls.

Recently, various methods using biological fluids have been developed for diagnosing PD: oligomerized and phosphorylated a-synuclein in CSF (AUC = approximately 0.67–0.82)^47^; caffeine and its metabolites in blood (AUC = 0.78–0.98)^48^; polyamines such as spermine and spermidine in blood (AUC = 0.87–0.98)^49^; microRNAs (miRNAs), miR-137 and miR-124, recovered from plasma (AUC = 0.707 and 0.709, respectively)^50^; and kynurenine in urine (AUC = 0.776)^51^. The P450 inhibition assay is easier to perform and is faster than other assays because this assay does not require pretreatment, such as purification of exosomes, and it involves a single enzymatic reaction. The P450 inhibition assay (AUC = 0.740–0.910) is a novel assay based on the alteration of expression of P450s in patients with PD. It has been demonstrated that the use of multiple biomarkers can provide a more precise diagnosis for many types of cancer^52–54^. Thus, this assay may compensate for any limitations of other assays and contribute to a more precise diagnosis. Furthermore, this is the first study to show that a P450 inhibition assay can be applied to human diagnosis. The P450 inhibition assay may be applicable to any other disease that shows altered expression levels of P450s such as cancer^9^ and cardiovascular disease^10^.

In this study, we revealed that the P450 inhibition assay could discriminate between rotenone-administered PD model rats and control rats. Moreover, this assay could discriminate between sera recovered from patients with PD and healthy volunteers. However, this study had a few limitations. This study was a case–control study; therefore, extensive studies such as cohort studies should be performed to evaluate this assay in a diagnosis system for PD. In addition, we could not obtain data on the expression levels and genetic polymorphisms of P450s in patients with PD and healthy volunteers. To the best of our knowledge, there are few reports on the expression levels of P450s in patients with PD. Expression levels of CYP2E1 are increased in the brains of patients with PD^24^; however, the expression levels of other P450s are unknown. Moreover, the expression levels of P450s in other organs such as the liver, lungs, and intestines of patients with PD are still unknown. To further understand the mechanisms of the P450 inhibition assay, the P450 expression levels and genetic polymorphisms in patients with PD and healthy volunteers should be investigated. Taken together, this study preliminarily demonstrated the possibility of a novel diagnosis system, P450 inhibition assay, which can be used for PD diagnosis.

## Methods

### Chemicals

Isopropyl-1-thio-β-D-galactopyranoside (IPTG), 5-aminolevulinic acid hydrochloride (5-ALA), and radioimmunoprecipitation (RIPA) buffer were obtained from Nacalai Tesque (Kyoto, Japan). Isoflurane, corn oil, and ampicillin (ABPC) were obtained from FUJIFILM Wako Pure Chemical Corporation (Osaka, Japan). Rotenone was obtained from Tokyo Chemical Industry Co., Ltd. (Tokyo, Japan). Glucose-6-phosphate (G6P), glucose-6-phosphate dehydrogenase (G6PDH), and nicotinamide adenine dinucleotide phosphate·4H_2_O (NADPH) were obtained from Oriental Yeast Company (Tokyo, Japan). Vivid® fluorescent substrates, dibenzyl-8-methyl-fluorescein (DBOMF), 7-benzyloxy-methyloxy-3-cyanocoumarin (BOMCC), and 7-ethoxy-methyloxy-3-cyanocoumarin (EOMCC), were obtained from Thermo Fisher Scientific (Waltham, MA, USA).

### Clinical samples

Human serum samples were purchased from BioIVT LLC (Westbury, NY, USA). In total, 20 serum samples of patients with PD (age, 64.9 ± 10.6 years) and 20 serum samples from healthy volunteers (age, 58.9 ± 14.6 years) were subjected to P450 inhibition assays (Supplementary Table S4). These clinical samples were collected by BioIVT LCC under protocols approved by the IRB of the WCG institute (Puyallup, WA, USA).

### Animal experiments

To develop Parkinson’s model animals, we adopted the rotenone-administered rat model, a well-established PD animal model^29–31^. Eight-week-old male Sprague–Dawley rats (n = 17) were purchased from Japan SLC (Shizuoka, Japan). The rats were housed at room temperature (23 ± 2 °C) with 50 ± 10% relative humidity at a 14:10 h light: dark cycle (light, 7:00 to 21:00) and were fed rodent diet DC-8 (CLEA Japan, Inc., Tokyo, Japan). On the day of the experiment, the rats received intraperitoneal administration of rotenone (3 mg/kg) dissolved in corn oil. Control rats received an intraperitoneal administration of corn oil only. Administration of rotenone was repeated for five d. After five d of serial administration of rotenone, the motor ability was assessed using the open field test as described below. This study was approved by the Institutional Animal Care and Use Committee (IACUC) of Kobe University (Approval number: 2020-02-01) and was performed according to the Kobe University Animal Experimentation Regulations and Animal Research: Reporting of In Vivo Experiments (ARRIVE) guidelines.

### Open field test

Open field tests were performed using a box with an open top (90 cm × 90 cm × 40 cm), and the floor was divided by black lines into 64 equal squares (8 × 8). Rats were introduced into the center of the box and allowed to explore the area freely for five min. The total distance traveled was evaluated by counting the number of lines crossed, and rearing frequency was measured.

### Tissue processing

Two days after the last rotenone administration, the rats were anesthetized via inhalation of isoflurane (3–5%), and whole blood was collected. Blood was incubated at 4 °C for 24 h, followed by centrifugation at 3000×*g* for 30 min, and serum samples were obtained. After collection of whole blood, the rats were transcardially perfused with 100 mL of phosphate-buffered saline (PBS). Brains were dissected and the striatum was collected. The liver was then removed and collected. These samples were frozen in liquid nitrogen and stored at − 80 °C until use.

### Gene expression analyses via RT-qPCR

Liver tissues harvested from rats were homogenized using a frozen sample crusher TK-AM5 (Tokken Inc., Chiba, Japan), and total RNA was isolated using NucleoSpin® RNA (Macherey–Nagel, Düren, Germany). Reverse transcription was performed using the ReverTra Ace® qPCR RT Kit (Toyobo Co., Ltd., Osaka, Japan). Quantitative PCR analysis was performed using KOD FX Neo (Toyobo) and SYBR Green I Nucleic Acid Gel Stain (Takara Bio, Shiga, Japan) on a CFX Connect system (Bio-Rad Laboratories Inc., Hercules CA, USA). The primers used in this study are listed in Supplementary Table S5. PCR amplification conditions consisted of 40 cycles at 95 °C for 10 s and 60 °C for 30 s. The genes were quantified by applying standard curve methods. For instance, to quantify the copy numbers of the P450s and b-actin, each gene fragment was prepared by PCR, and the amount was quantified by measuring absorbance at 260 nm. The copy number of each product was calculated, and a standard curve was constructed for each gene. Expression levels of each gene were normalized to those of b-actin.

### Immunoblotting analysis

Striatum samples recovered from the rats were homogenized using frozen sample crusher TK-AM5 (Tokken) and resolved in RIPA buffer containing a protease inhibitor cocktail. After incubation on ice for 1 h, the samples were centrifuged (10,000 × *g*, 4 °C, 10 min) and the supernatant was collected. Protein concentrations were determined using a DC Protein Assay (Bio-Rad Laboratories) according to the manufacturer’s instructions. Samples were resolved via 10% sodium dodecyl sulfate–polyacrylamide gel electrophoresis and transferred to a polyvinylidene fluoride (PVDF) membrane (ATTO Corporation, Tokyo, Japan). The PVDF membrane was blocked using a Blocking One (Nacalai Tesque), followed by incubation with primary antibodies at 23 °C for 1 h. The following antibodies were used: anti-TH antibody (Merck KGaA, Darmstadt, Germany) and anti-beta actin antibody (mAbcam 8226; Abcam, Cambridge, United Kingdom). Subsequently, the membranes were incubated with secondary antibodies, KPL peroxidase-labeled affinity-purified antibody to mouse IgG (H + L) (Seracare Life Sciences Inc., Milford, MA, USA). Target proteins were visualized using Chemi-Lumi One Super (Nacalai Tesque), and signals were detected using ChemiDoc™ Touch (Bio-Rad Laboratories). The signal intensity of the bands was quantified using Image J software (National Institutes of Health, Bethesda, MD, USA).

### Rotenone quantification in rat sera via HPLC

Rotenone was extracted from 0.5 mL of serum by mixing with an equal volume of ethyl-acetate and then centrifuged at 15,000×*g* for 20 min (Himac CF15R, Eppendorf Himac Technologies Co., Ltd., Ibaraki, Japan). 0.3 mL of the organic phase was transferred to a new collection tube and evaporated to dryness at 23 °C. The residue was dissolved in 0.15 mL of methanol. To create a standard curve of the amount of recovered rotenone from serum, 1,000–0.005 μg/mL of rotenone was added to pooled human serum purchased from Seracare Life Sciences Inc. (Milford, MA, USA) and the extracted rotenone as described above. HPLC analyses were performed using the Hitachi D-7000 HPLC system (Hitachi, Ltd., Tokyo, Japan). Chromatographic separation was performed on TSKgel ODS-80Ts column, 150 mm × 4.6 mm i.d., 5 μm particle size, (Tosoh Corporation, Tokyo, Japan) with 70:30 (v/v) methanol:H_2_O as the mobile phase. The injection volume was 10 μL, and the mobile phase flow rate was 1.0 mL/min. Rotenone was detected by measuring the absorbance at 254 nm.

### Preparation of *E. coli* membrane fractions expressing human P450 and P450 reductase

*E. coli* strains harboring a plasmid encoding human P450 (CYP1A1, CYP1A2, CYP2A13, CYP2B6, CYP2C8, CYP2C9, CYP2C18, CYP2E1, CYP3A4, or CYP3A5) and P450 reductase^15^ were grown in 3 mL of LB medium (1% tryptone, 0.5% yeast extract, and 1% NaCl) containing 50 μg/mL of ABPC for 18 h at 37 °C. The culture (1 mL) was inoculated into 100 mL of TB medium (1.2% [w/v] tryptone, 2.4% [w/v] yeast extract, 0.4% [w/v] glycerol, and 100 mM potassium phosphate buffer; pH 7.4) containing 50 μg/mL of ABPC and incubated at 37 °C until the optical density at 600 nm reached 0.5. Subsequently, IPTG and 5-ALA were added at concentrations of 1 mM and 0.5 mM, respectively, and the cells were incubated for 24 h at 25 °C for CYP2A13, CYP2C8, CYP2C9, CYP2C18, CYP2E1, CYP3A4, and CYP3A5; for 40 h at 25 °C for CYP2B6; and for 24 h at 30 °C for CYP1A1 and CYP1A2. Cells were harvested via centrifugation (5000×*g*, 10 min, 4 °C) and washed with 20 mL of stock buffer (100 mM potassium phosphate buffer [pH 7.4], 1 mM ethylenediaminetetraacetic acid, and 20% glycerol). The cells were resuspended in 20 mL of stock buffer and disrupted using an ultrasonicator VP-300 N (Taitech Co., Saitama, Japan). Cells were centrifuged (5000×*g*, 15 min, 4 °C) and supernatants were collected, followed by ultracentrifugation (100,000 × *g*, 70 min, 4 °C). Pellets containing membrane fractions were resuspended in stock buffer and stored at − 80 °C. Protein concentration in the membrane fraction was determined using Bradford’s method and a protein assay kit (Bio-Rad). To determine the amount of P450 proteins in each membrane sample, reduced carbon monoxide (CO)-difference spectra were measured using a Hitachi UV visible spectrophotometer U-3300 (Hitachi, Tokyo, Japan), as previously described^55^. The P450 hemoprotein contents in *E. coli* suspensions were determined using an extinction coefficient of 91.1 mM^-1^ cm^-1^^56^ (Supplementary Fig. S4). Measurements of protein concentrations and the amount of P450 proteins are described in Supplementary Table S6. Membrane fractions for CYP2D6 were purchased from Cypex Ltd. (Dundee, Scottland, UK). The concentration of each membrane fraction was optimized to produce an equal amount of fluorescent substances.

### P450 inhibition assay using human serum samples

The P450 inhibition assay was performed as previously described^15^. In brief, 10 μL of 200 mM potassium phosphate buffer (pH 7.4), 4 μL of NADPH regeneration buffer (100 mM potassium phosphate buffer [pH 7.4], 10 mM G6P, 2 mM NADPH, 2 units/mL G6PDH), and 6 μL of diluted P450-containing membrane fraction were added to a 384-well plate. Dilution factors of each membrane of the fraction are described in Supplementary Table S7. Subsequently, 16 μL of serum diluted 1:40 with PBS was added to the 384-well plate, and the plate was incubated at 37 °C for 30 min. The concentrations of the serum samples were optimized in order not to saturate the inhibition rate. PBS was added instead of diluted serum in the control group. After 30 min of incubation, 4 μL of Vivid® fluorescent substrates, DBOMF, BOMCC, and EOMCC, were added to the plate. DBOMF was used as a substrate for CYP2C8 and CYP3A4. BOMCC was used as a substrate for CYP2B6, CYP2C9, and CYP3A5. EOMCC was used as a substrate for CYP1A1, CYP1A2, CYP2A13, CYP2C18, CYP2C19, and CYP2C19. The plate was incubated for 40 min at 37 °C, and fluorescence values were measured using an automatic plate reader MTP-810Lab (Corona Electric, Ibaraki, Japan) at an excitation wavelength of 492 nm and an emission wavelength of 530 nm for DBOMF, and at 405 nm and 450 nm for both BOMCC and EOMCC. Inhibition rates of metabolic activity were assessed by comparing the fluorescence intensity of samples containing sera and control samples not containing sera.

### P450 inhibition assay using sera from rotenone-administered PD model rats

P450 inhibition assays using rat sera were performed in the same manner as the assay using human sera with certain modifications. The solutions used in this assay were dispensed using an automated liquid handler epMotion® 5073 (Eppendorf, Hamburg, Germany). The serum dilution ratio was modified as follows: 1:10 for CYP1A1, CYP1A2, CYP2B6, CYP2C19, and CYP2E1; 1:40 for CYP2C8, CYP2C9, CYP3A4, and CYP3A5; and 1:100 for CYP2A13 and CYP2C18. The concentrations of the serum samples were optimized in order not to saturate the inhibition rate.

### Statistical analysis

Values are presented as the mean ± standard error of the mean. Differences between measurements and groups were compared using Welch’s t-test. ROC curve analysis, Pearson's correlation coefficient, Spearman’s rank correlation coefficient, and logistic regression analysis were performed using JMP 13 for Windows software program (SAS Institute Inc., Cary, NC, USA). Cut-off values obtained from ROC curve analysis were determined according to the Youden Index^57^. A two-tailed p-value of < 0.05 was considered significant.

## Supplementary Information

Supplementary Information 1.

Supplementary Information 2.

Supplementary Information 3.

Supplementary Information 4.

Supplementary Information 5.

Supplementary Information 6.

Supplementary Information 7.

Supplementary Information 8.

## Data Availability

Correspondence and requests for materials should be addressed to Hiromasa Imaishi.

## References

[1] Vos T (2017). Global, regional, and national incidence, prevalence, and years lived with disability for 328 diseases and injuries for 195 countries, 1990–2016: A systematic analysis for the Global Burden of Disease Study 2016. Lancet.

[2] Obeso JA, Rodriguez-Oroz MC, Stamelou M, Bhatia KP, Burn DJ (2014). The expanding universe of disorders of the basal ganglia. Lancet.

[3] Block ML, Zecca L, Hong JS (2007). Microglia-mediated neurotoxicity: uncovering the molecular mechanisms. Nat. Rev. Neurosci..

[4] Qian L, Flood PM, Hong JS (2010). Neuroinflammation is a key player in Parkinson’s disease and a prime target for therapy. J. Neural Transm. (Vienna).

[5] Zanger UM, Schwab M (2013). Cytochrome P450 enzymes in drug metabolism: regulation of gene expression, enzyme activities, and impact of genetic variation. Pharmacol. Ther..

[6] Guengerich FP (2008). Cytochrome P450 and chemical toxicology. Chem. Res. Toxicol..

[7] Rendic S (2002). Summary of information on human CYP enzymes: human P450 metabolism data. Drug Metab. Rev..

[8] de Jong LM, Jiskoot W, Swen JJ, Manson ML (2020). Distinct effects of inflammation on cytochrome P450 regulation and drug metabolism: lessons from experimental models and a potential role for pharmacogenetics. Genes.

[9] Oyama T (2004). Expression of cytochrome P450 in tumor tissues and its association with cancer development. Front. Biosci..

[10] Elbekai RH, El-Kadi AO (2006). Cytochrome P450 enzymes: central players in cardiovascular health and disease. Pharmacol. Ther..

[11] Matzke GR, Frye RF, Early JJ, Straka RJ, Carson SW (2000). Evaluation of the influence of diabetes mellitus on Antipyrine metabolism and CYP1A2 and CYP2D6 activity. Pharmacotherapy.

[12] Wang Z (2003). Diabetes mellitus increases the in vivo activity of cytochrome P450 2E1 in humans. Br. J. Clin. Pharmacol..

[13] Chaluvadi MR, Nyagode BA, Kinloch RD, Morgan ET (2009). TLR4-dependent and-independent regulation of hepatic cytochrome P450 in mice with chemically induced inflammatory bowel disease. Biochem. Pharmacol..

[14] Kusunoki Y (2014). Hepatic early inflammation induces downregulation of hepatic cytochrome P450 expression and metabolic activity in the dextran sulfate sodium-induced murine colitis. Eur. J. Pharm. Sci..

[15] Yamamoto R, Muroi K, Imaishi H (2018). Serum derived from ulcerative colitis mouse changes the metabolism of the fluorescent substrate by P450 depending on the degree of disease progression. Chem. Biol. Interact..

[16] Marks BD (2003). High-Throughput screeening assays for CYP2B6 metabolism and inhibition usuing fluorogenic vivid substrates. AAPS PharmSci.

[17] Marks BD, Thompson DV, Goossens TA, Trubetskoy OV (2004). High-throughput screening assays for the assessment of CYP2C9* 1, CYP2C9* 2, and CYP2C9* 3 metabolism using fluorogenic Vivid® substrates. J. Biomol. Screen..

[18] Trubetskoy OV, Gibson JR, Marks BD (2005). Highly miniaturized formats for in vitro drug metabolism assays using Vivid® fluorescent substrates and recombinant human cytochrome P450 enzymes. J. Biomol. Screen..

[19] Tamaki S, Imaishi H (2020). Inhibitory effects of type 2 diabetes serum components in P450 inhibition assays can potential diagnose asymptomatic diabetic mice. Drug Metab. Pharmacokinet..

[20] Dolatshahi, M., Ranjbar Hameghavandi, M. H., Sabahi, M. & Rostamkhani, S. Nuclear factor‐kappa B (NF-κB) in pathophysiology of Parkinson disease: diverse patterns and mechanisms contributing to neurodegeneration. *Eur. J. Neurosci.* (2021). 10.1111/ejn.15242 33884689

[21] Mogi M (1994). Tumor necrosis factor-a (TNF-a) increases both in the brain and in the cerebrospinal fluid from parkinsonian patients. Neurosci. Lett..

[22] Blum-Degen D (1995). Interleukin-1β and interleukin-6 are elevated in the cerebrospinal fluid of Alzheimer's and de novo Parkinson's disease patients. Neurosci. Lett..

[23] Brodacki B (2008). Serum interleukin (IL-2, IL-10, IL-6, IL-4), TNFα, and INFγ concentrations are elevated in patients with atypical and idiopathic parkinsonism. Neurosci. Lett..

[24] Kaut O, Schmitt I, Wüllner U (2012). Genome-scale methylation analysis of Parkinson's disease patients' brains reveals DNA hypomethylation and increased mRNA expression of cytochrome P450 2E1. Neurogenetics.

[25] Wójcikowski J, Daniel WA (2009). The brain dopaminergic system as an important center regulating liver cytochrome P450 in the rat. Expert Opin. Drug Metab. Toxicol..

[26] Miksys S, Tyndale RF (2013). CCNP Heinz Lehmann Award paper: cytochrome P450-mediated drug metabolism in the brain. J. Psychiatry Neurosci. JPN.

[27] Ur Rasheed MS, Mishra AK, Singh MP (2017). Cytochrome P450 2D6 and Parkinson's disease: Polymorphism, metabolic role, risk and protection. Neurochem. Res..

[28] Cordato DJ, Chan DK (2004). Genetics and Parkinson’s disease. J. Clin. Neurosci..

[29] Tieu K (2011). A guide to neurotoxic animal models of Parkinson’s disease. Cold Spring Harb. Perspect. Med..

[30] Bové J, Perier C (2012). Neurotoxin-based models of Parkinson's disease. Neuroscience.

[31] Miyazaki I, Asanuma M (2020). The rotenone models reproducing central and peripheral features of Parkinson’s disease. Neuroscience.

[32] Fawcett T (2006). An introduction to ROC analysis. Pattern Recognit. Lett..

[33] Hoehn MM, Yahr MD (1967). Parkinsonism: onset, progression, and mortality. Neurology.

[34] Radad K (2019). Rotenone: From modelling to implication in Parkinson’s disease. Folia Neuropathol..

[35] Anderson KE, Kappas A (1991). Dietary regulation of cytochrome P450. Annu. Rev. Nutr..

[36] Nelson DR (2009). The cytochrome P450 homepage. Hum. Genomics.

[37] Lewis DF, Ioannides C, Parke DV (1998). Cytochromes P450 and species differences in xenobiotic metabolism and activation of carcinogen. Environ. Health Perspect..

[38] Kot M, Daniel WA (2011). Cytochrome P450 is regulated by noradrenergic and serotonergic systems. Pharmacol. Res..

[39] Martínez C (2000). Modulation of midazolam 1-hydroxylation activity in vitro by neurotransmitters and precursors. Eur. J. Clin. Pharmacol..

[40] Anna Haduch A, Bromek E, Daniel WA (2013). Role of brain cytochrome P450 (CYP2D) in the metabolism of monoaminergic neurotransmitters. Pharmacol. Rep..

[41] Nehlig A (2018). Interindividual differences in caffeine metabolism and factors driving caffeine consumption. Pharmacol. Rev..

[42] Sontag TJ, Parker RS (2002). Cytochrome P450 ω-hydroxylase pathway of tocopherol catabolism: Novel mechanism of regulation of vitamin E status. J. Biol. Chem..

[43] Birringer M, Drogan D, Brigelius-Flohe R (2001). Tocopherols are metabolized in HepG2 cells by side chain ω-oxidation and consecutive β-oxidation. Free Radic. Biol. Med..

[44] Goldstein DS, Holmes C, Sharabi Y (2012). Cerebrospinal fluid biomarkers of central catecholamine deficiency in Parkinson’s disease and other synucleinopathies. Brain.

[45] Molina JA (1997). Decreased cerebrospinal fluid levels of neutral and basic amino acids in patients with Parkinson's disease. J. Neurol. Sci..

[46] Buhmann C (2004). Plasma and CSF markers of oxidative stress are increased in Parkinson's disease and influenced by antiparkinsonian medication. Neurobiol. Dis..

[47] Majbour NK (2016). Oligomeric and phosphorylated alpha-synuclein as potential CSF biomarkers for Parkinson’s disease. Mol. Neurodegener..

[48] Fujimaki M (2018). Serum caffeine and metabolites are reliable biomarkers of early Parkinson disease. Neurology.

[49] Saiki S (2019). A metabolic profile of polyamines in Parkinson disease: A promising biomarker. Ann. Neurol..

[50] Li N (2017). Plasma levels of miR-137 and miR-124 are associated with Parkinson’s disease but not with Parkinson’s disease with depression. Neurol. Sci..

[51] Bai JH, Zheng YL, Yu YP (2021). Urinary kynurenine as a biomarker for Parkinson’s disease. Neurol. Sci..

[52] Uribarri M (2014). A new biomarker panel in bronchoalveolar lavage for an improved lung cancer diagnosis. J. Thorac. Oncol..

[53] Fung KY (2015). Blood-based protein biomarker panel for the detection of colorectal cancer. PLoS ONE.

[54] Severi G (2014). A three-protein biomarker panel assessed in diagnostic tissue predicts death from prostate cancer for men with localized disease. Cancer Med..

[55] Imaishi H, Matsuo S, Swai E, Ohkawa H (2000). CYP78A1 preferentially expressed in developing inflorescences of Zea mays encoded a cytochrome P450-dependent lauric acid 12-monooxygenase. Biosci. Biotechnol. Biochem..

[56] Omura T, Sato R (1964). The carbon monoxide-binding pigment of liver microsomes: I. Evidence for its hemoprotein nature. J. Biol. Chem..

[57] Youden WJ (1950). Index for rating diagnostic tests. Cancer.

