# Supplementary material for: Diagnosis of Parkinson's disease by investigating the inhibitory effect of serum components on P450 inhibition assay
Source: Sci Rep. 2022 Apr 22;12:6622. doi: 10.1038/s41598-022-10528-x (PMC9033851; doi:10.1038/s41598-022-10528-x)
Supplement: Supplementary file 1 — Supplementary Information 1. [file 41598_2022_10528_MOESM1_ESM.pdf]

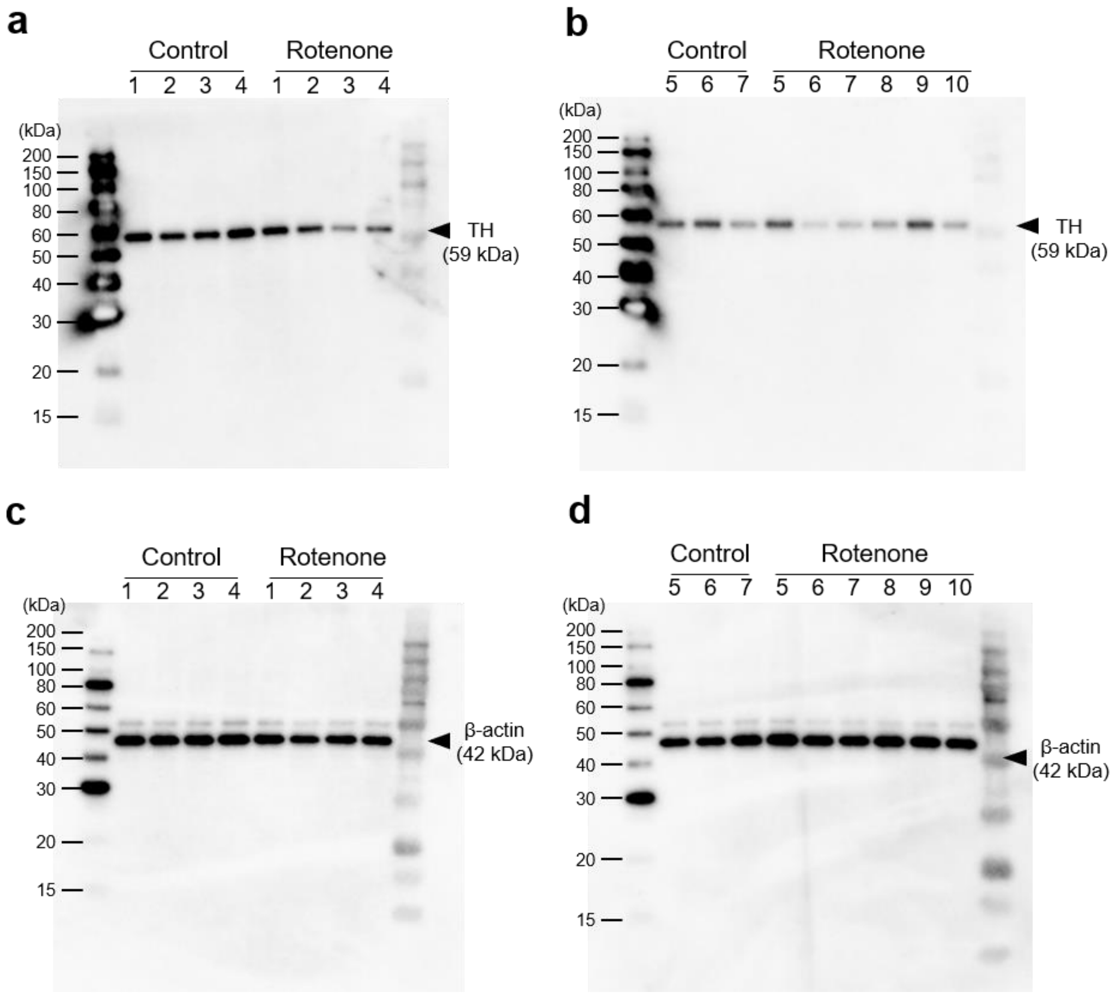

Supplementary Fig. S1. Raw data of the immunoblotting analysis (Fig. 1c). Expression levels of TH and β-actin in the rat striatum were analyzed via immunoblotting analyses using anti-TH antibodies (a and b) and anti-β-actin antibodies (c and d). YesBlot™ Western Marker I (SMOBIO Technology, Inc., Hsinchu, Taiwan) (left lane) and Precision Plus Protein™ Dual Color Standards (Bio-Rad Laboratories Inc., Hercules CA, USA) (right lane) were used as molecular markers. TH, tyrosine hydroxylase

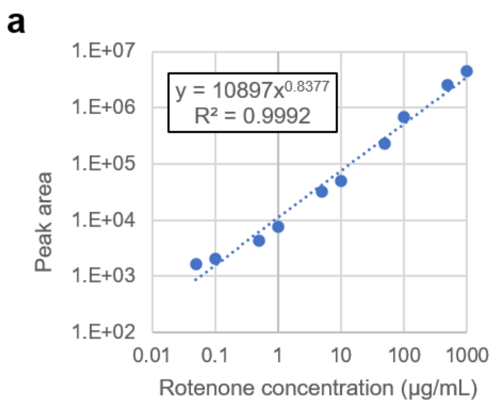

b

Rotenone Concentration (µg/ml)	Peak area
1000	4.56E+06
500	2.53E+06
100	6.75E+05
50	2.29E+05
10	5.02E+04
5	3.24E+04
1	7.43E+03
0.5	4.25E+03
0.1	2.09E+03
0.05	1.62E+03
0.01	N.D.
0.005	N.D.

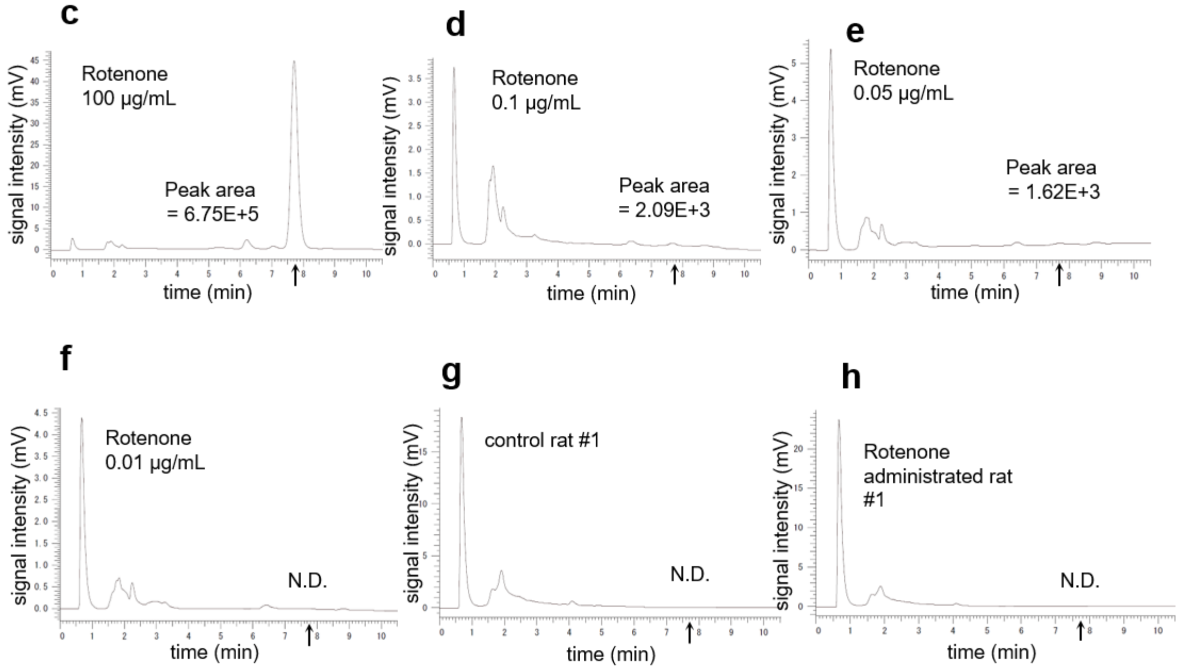

Supplementary Fig. S2. High-performance liquid chromatography (HPLC) analyses for quantification of rotenone in the sera recovered from rats. (a) and (b). To create the standard curve of the amount of recovered rotenone from serum, 1,000–0.005 µg/mL of rotenone was added to pooled human serum. Chromatograms of representative results of HPLC analyses of (c) rotenone recovered from pooled serum containing 100 µg/mL of rotenone, (d) rotenone recovered from pooled serum containing 0.1 µg/mL of rotenone, (e) rotenone recovered from pooled serum containing 0.05 µg/mL of rotenone, (f) rotenone recovered from pooled serum containing 0.01 µg/mL of rotenone, (g) rotenone recovered from the serum of control rat #1, and (h) rotenone recovered from the serum of rotenone-administrated rat #1 were presented. The arrows indicate the retention time of approximately 7.7 min, through which the peak of rotenone was detected.

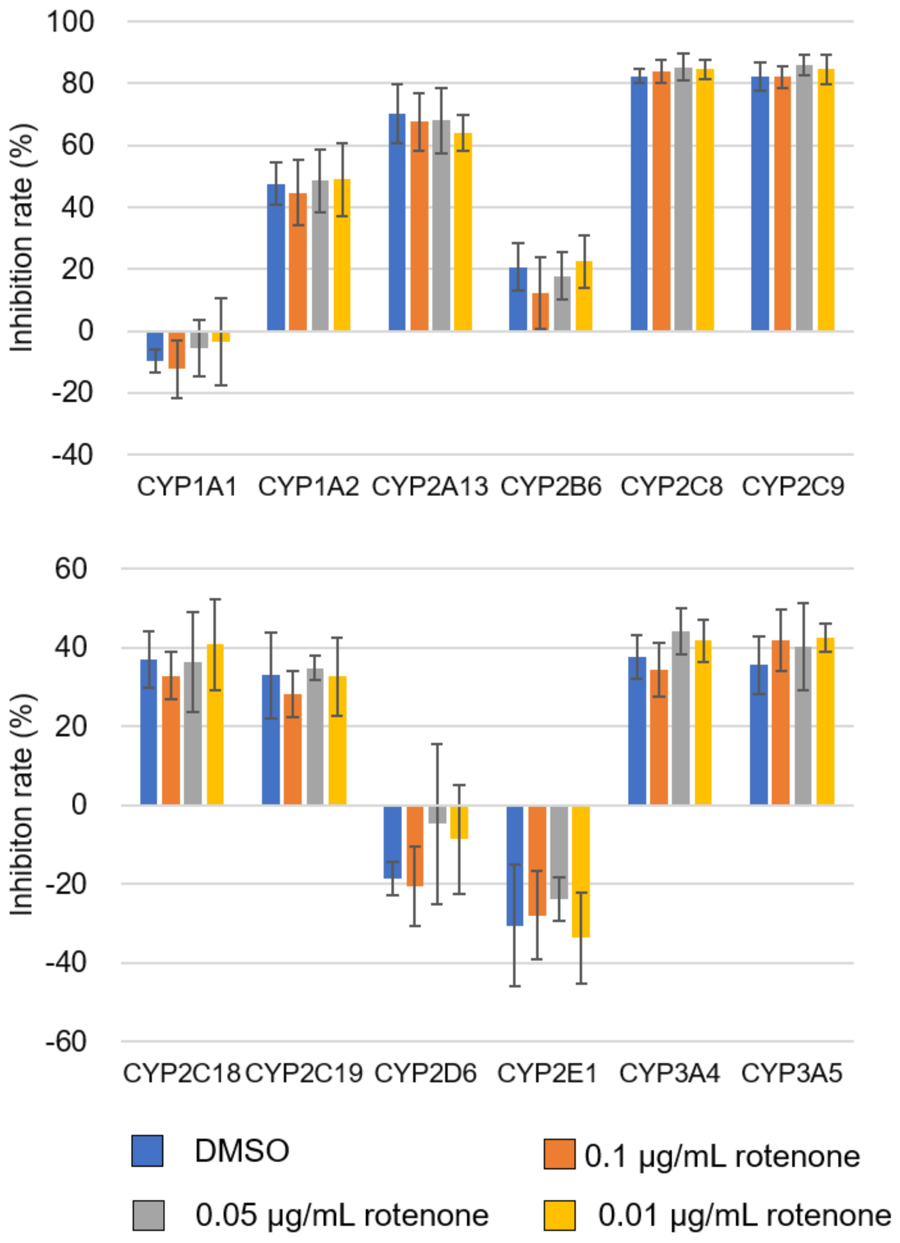

Supplementary Fig. S3. P450 inhibition assay using sera containing 0.1, 0.05, and 0.01 μg/mL rotenone. Differences between sera to which DMSO was added and sera containing various rotenone concentrations were evaluated using the Tukey–Kramer test ($p < 0.05$). No significant differences were observed between the groups. DMSO, dimethyl sulfoxide.

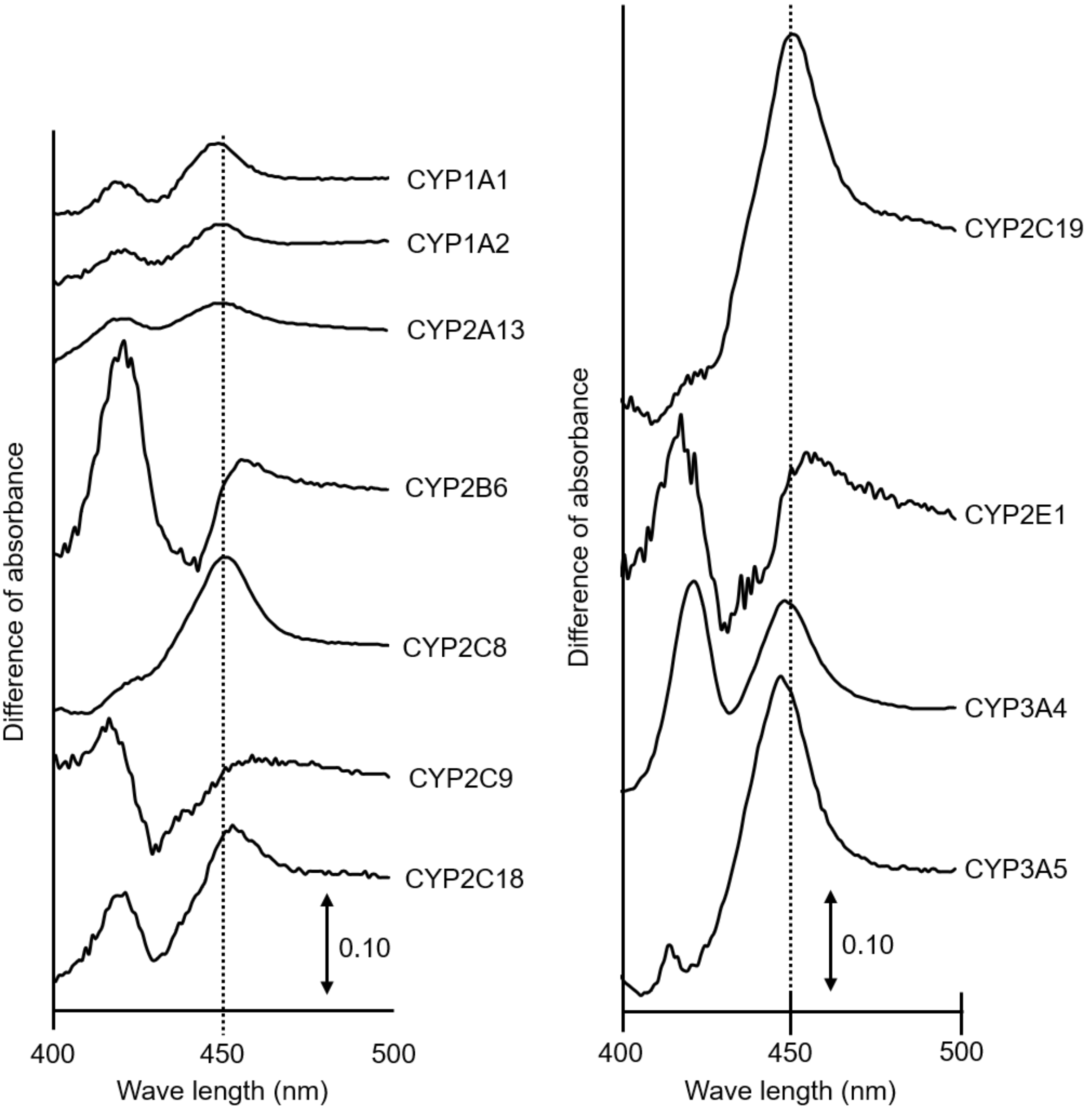

Supplementary Fig. S4. Reduced CO-difference spectra of each P450-containing membrane fraction. Differences in absorbance ranging from 400–500 nm for each P450 are represented in the graph. The two direction arrows demonstrate the scale of difference in absorbance (0.10).
