# Supplementary material for: Diagnosis of Parkinson's disease by investigating the inhibitory effect of serum components on P450 inhibition assay
Source: Sci Rep. 2022 Apr 22;12:6622. doi: 10.1038/s41598-022-10528-x (PMC9033851; doi:10.1038/s41598-022-10528-x)
Supplement: Supplementary file 2 — Supplementary Information 2. [file 41598_2022_10528_MOESM2_ESM.pdf]

Supplementary table 1. Clinical samples used in this study

		HV (n = 20)	PD (n = 20)	AD (n=19)	T2D (n=10)
age[†]		58.9 ± 14.6	64.9 ± 10.6	67.4 ± 12.2	54,2 ± 13.0
sex	Male	n = 10	n = 10	n = 9	n = 5
	Female	n = 10	n = 10	n = 10	n = 5
Stage of the Hohen & Yahr scale	0	n = 20	-	n = 19	n =10
	1	-	n = 8	-	-
	1.6	-	n = 1	-	-
	2	-	n = 2	-	-
	2.5	-	n=1	-	-
	3	-	n = 2	-	-
	N/A	-	n = 4	-	-

†: data were represented average ± SD
