# Supplementary material for: Diagnosis of Parkinson's disease by investigating the inhibitory effect of serum components on P450 inhibition assay
Source: Sci Rep. 2022 Apr 22;12:6622. doi: 10.1038/s41598-022-10528-x (PMC9033851; doi:10.1038/s41598-022-10528-x)
Supplement: Supplementary file 3 — Supplementary Information 3. [file 41598_2022_10528_MOESM3_ESM.pdf]

Supplementary table 2. Primers used in this study

Oligoname	sequence 5'- -3'
Rat Cyp1a1 fwd	TCCTGGAGACCTTCCGACAT
Rat Cyp1a1 rev	ACCTGCCACTGGTTCACAAA
Rat Cyp1a2 fwd	CACAGCACAACGAGGGACA
Rat Cyp1a2 rev	TCTGGGCGGAACACAAAG
Rat Cyp2b1 fwd	GGGAAAGAGGAGTGTGGAAGAA
Rat Cyp2b1 rev	GAGCAGATGATGTTGGCTGTG
Rat Cyp2b2 fwd	GGGAAAGAGGAGTGTGGAAGAA
Rat Cyp2b2 rev	GGAGAATGAACTTAGGAGGGAAA
Rat Cyp2c6 fwd	CTGTTGCTCCTGCTGAAGTGTCC
Rat Cyp2c6 rev	CCGCATGTGGCAGGTTGGTAG
Rat Cyp2c11 fwd	AGCTTGGTGGCTACTGTAACTGAC
Rat Cyp2c11 rev	CAGCAGCAGCAGGAGTCCATAC
Rat Cyp2d2 fwd	GCCTGGAAGCCTGTAGTTGTGATC
Rat Cyp2d2 rev	TCGTCTCTGCTCTCGCCACTC
Rat Cyp2e1 fwd	GGAAGGATGTGCGGAGGTT
Rat Cyp2e1 rev	CAGAAATGTGGGGTCAAAAGG
Rat Cyp3a1 fwd	ATGATTCCATCTTATGCTCTTCACC
Rat Cyp3a1 rev	CTGCCCTTGTTCTCCTTGCT
Rat Cyp3a2 fwd	GATCCTTTTGTGGAGAAAACCAAG
Rat Cyp3a2 rev	TTGGGGTGAGGAATGGAAAG
Rat β-actin fwd	GGAGATTACTGCCCTGGCTCCTA
Rat β-actin rev	GACTCATCGTACTCCTGCTTGCTG
