# Supplementary material for: Diagnosis of Parkinson's disease by investigating the inhibitory effect of serum components on P450 inhibition assay
Source: Sci Rep. 2022 Apr 22;12:6622. doi: 10.1038/s41598-022-10528-x (PMC9033851; doi:10.1038/s41598-022-10528-x)
Supplement: Supplementary file 4 — Supplementary Information 4. [file 41598_2022_10528_MOESM4_ESM.pdf]

Supplementary table 3. Fluorescent substrates and dilution factors of each P450-containing membrane fraction.

P450 species	Vivid® substrate	Dilution factor
CYP1A1	EOMCC	485
CYP1A2	EOMCC	132
CYP2A13	EOMCC	120
CYP2B6	BOMCC	258
CYP2C8	DBOMF	664
CYP2C9	BOMCC	5.5
CYP2C18	EOMCC	5.5
CYP2C19	EOMCC	95.7
CYP2D6	EOMCC	38.8
CYP2E1	EOMCC	1
CYP3A4	DBOMF	1354
CYP3A5	BOMCC	41

EOMCC: 7-ethoxy-methyloxy-3-cyanocoumarin

BOMCC: 7-benzyloxy-methyloxy-3-cyanocoumarin

DBOMF: dibenzyl-8-methyl-fluorescein
