# Supplementary material for: Diagnosis of Parkinson's disease by investigating the inhibitory effect of serum components on P450 inhibition assay
Source: Sci Rep. 2022 Apr 22;12:6622. doi: 10.1038/s41598-022-10528-x (PMC9033851; doi:10.1038/s41598-022-10528-x)
Supplement: Supplementary file 5 — Supplementary Information 5. [file 41598_2022_10528_MOESM5_ESM.pdf]

Supplementary table 4. P450 expression levels and protein concentrations of each P450-containing membrane fraction

P450 species	P450 expression level (pmol/mg of membrane protein)	Protein concentration (mg/mL of membrane fraction)
CYP1A1	44.4	8.65
CYP1A2	20.1	9.28
CYP2A13	48.8	5.40
CYP2B6	31.8	9.68
CYP2C8	159.6	5.71
CYP2C9	37.8	4.36
CYP2C18	348.6	6.42
CYP2C19	72.2	5.47
CYP2D6	172.0	16.30
CYP2E1	59.3	4.44
CYP3A4	148.1	7.86
CYP3A5	307.8	7.17
