# Supplementary material for: Diagnosis of Parkinson's disease by investigating the inhibitory effect of serum components on P450 inhibition assay
Source: Sci Rep. 2022 Apr 22;12:6622. doi: 10.1038/s41598-022-10528-x (PMC9033851; doi:10.1038/s41598-022-10528-x)
Supplement: Supplementary file 6 — Supplementary Information 6. [file 41598_2022_10528_MOESM6_ESM.pdf]

Supplementary table 5. Diagnostic values of each P450 inhibition rate for rotenone administrated rats.

Factor	Cut off value*	Sensitivity (%)	Specificity (%)	Accuracy (%)	PPV (%)	NPV (%)
CYP1A1	10.0	60.0<br>(6/10)	100.0<br>(7/7)	76.5<br>(13/17)	100.0<br>(6/6)	63.6<br>(7/11)
CYP1A2	49.9	40.0<br>(4/10)	71.4<br>(5/7)	52.9<br>(9/17)	66.7<br>(4/6)	45.5<br>(5/11)
CYP2A13	77.1	90.0<br>(9/10)	85.7<br>(6/7)	88.2<br>(15/17)	90.0<br>(9/10)	85.7<br>(6/7)
CYP2B6	-22.6	50.0<br>(5/10)	71.4<br>(5/7)	58.8<br>(10/17)	71.4<br>(5/7)	50.0<br>(5/10)
CYP2C8	84.8	30.0<br>(3/10)	100.0<br>(7/7)	58.8<br>(10/17)	100.0<br>(3/3)	50.0<br>(7/14)
CYP2C9	50.9	10.0<br>(1/10)	100.0<br>(7/7)	47.1<br>(8/17)	100.0<br>(1/1)	43.8<br>(7/16)
CYP2C18	44.2	70.0<br>(7/10)	100.0<br>(7/7)	82.4<br>(14/17)	100.0<br>(7/7)	70.0<br>(7/10)
CYP2C19	-9.0	100.0<br>(10/10)	28.6<br>(2/7)	70.6<br>(12/17)	66.7<br>(10/15)	100.0<br>(2/2)
CYP2E1	-3.3	50.0<br>(5/10)	100.0<br>(7/7)	70.6<br>(12/17)	100.0<br>(5/5)	58.3<br>(7/12)
CYP3A4	32.7	60.0<br>(6/10)	100.0<br>(7/7)	76.5<br>(13/17)	100.0<br>(6/6)	63.6<br>(7/11)
CYP3A5	39.6	80.0<br>(8/10)	100.0<br>(7/7)	88.2<br>(15/17)	100.0<br>(8/8)	77.8<br>(7/9)

*: cut off values of inhibition rate of each P450s were determined according to Youden index.

PPV: positive predictive value

NPV: negative predictive value.
