# Supplementary material for: Diagnosis of Parkinson's disease by investigating the inhibitory effect of serum components on P450 inhibition assay
Source: Sci Rep. 2022 Apr 22;12:6622. doi: 10.1038/s41598-022-10528-x (PMC9033851; doi:10.1038/s41598-022-10528-x)
Supplement: Supplementary file 7 — Supplementary Information 7. [file 41598_2022_10528_MOESM7_ESM.pdf]

Supplementary table 6. Diagnostic values of each P450 inhibition rate for Parkinson's disease.

Factor	Cut off value*	Sensitivity (%)	Specificity (%)	Accuracy (%)	PPV (%)	NPV (%)
CYP1A1	11.3	80.0 (16/20)	65.0 (13/20)	72.5 (29/40)	70.0 (16/23)	76.5 (13/17)
CYP1A2	30.7	20.0 (4/20)	71.4 (20/20)	60.0 (24/40)	100.0 (4/4)	55.6 (20/36)
CYP2A13	95.5	100.0 (20/20)	20.0 (4/20)	60.0 (24/40)	55.6 (20/36)	100.0 (4/4)
CYP2B6	-2.5	85.0 (17/20)	55.0 (11/20)	65.0 (26/40)	60.7 (17/28)	75.0 (9/12)
CYP2C8	85.4	70.0 (14/20)	80.0 (16/20)	75.0 (30/40)	77.8 (14/18)	72.7 (16/22)
CYP2C9	86.5†	90.0 (18/20)	35.0 (7/20)	62.5 (25/40)	58.1 (18/31)	77.8 (7/9)
	87.1†	95.0 (19/20)	30.0 (6/20)	62.5 (25/40)	57.6 (19/33)	85.7 (6/7)
CYP2C18	77.1†	75.0 (15/20)	65.0 (13/20)	70.0 (28/40)	68.2 (15/22)	72.2 (13/18)
	76.1†	80.0 (16/20)	60.0 (12/20)	70.0 (28/40)	66.7 (16/24)	75.0 (12/16)
CYP2C19	11.7†	40.0 (8/20)	85.0 (17/20)	62.5 (25/40)	72.7 (8/11)	58.6 (17/29)
	4.1†	65.0 (13/20)	60.0 (12/20)	62.5 (25/40)	61.9 (13/21)	63.2 (12/19)
CYP2E1	21.1	30.0 (6/20)	95.0 (19/20)	70.6 (12/17)	85.7 (6/7)	57.6 (19/33)
CYP3A4	59.0	85.0 (17/20)	50.0 (10/20)	67.5 (27/40)	63.0 (17/27)	76.9 (10/13)
CYP3A5	67.3	100 (20/20)	50 (10/20)	75 (30/40)	66.7 (20/30)	100 (10/10)

*: cut off values of inhibition rate of each P450s were determined according to Youden index.

†: each cut off value showed same accuracy

PPV: positive predictive value

NPV: negative predictive value
