# Supplementary material for: Diagnosis of Parkinson's disease by investigating the inhibitory effect of serum components on P450 inhibition assay
Source: Sci Rep. 2022 Apr 22;12:6622. doi: 10.1038/s41598-022-10528-x (PMC9033851; doi:10.1038/s41598-022-10528-x)
Supplement: Supplementary file 8 — Supplementary Information 8. [file 41598_2022_10528_MOESM8_ESM.pdf]

Supplementary table 7. Diagnostic values of logistic regression model of inhibition rates of significantly changed P450s for Parkinson’s disea

Factor	Cut off value*	Sensitivity (%)	Specificity (%)	Accuracy (%)	PPV (%)	NPV (%)
CYP1A1<br>CYP2C8	0.6068	85.0<br>(17/20)	95.0<br>(19/20)	90.0<br>(36/40)	94.4<br>(17/18)	86.4<br>(19/22)
CYP1A1<br>CYP3A5	0.3159	100.0<br>(20/20)	50.0<br>(10/20)	75.0<br>(30/40)	66.7<br>(20/30)	100.0<br>(10/10)
CYP2C8<br>CYP3A5	0.3296	95.0<br>(19/20)	75.0<br>(15/20)	85.0<br>(34/40)	79.2<br>(19/24)	93.8<br>(15/16)
CYP1A1<br>CYP2C8<br>CYP3A5	0.4250	90.0<br>(18/20)	80.0<br>(16/20)	85.0<br>(34/40)	81.8<br>(18/22)	88.9<br>(16/18)

*: cut off values of logistic regression models were determined according to Youden index.

PPV: positive predictive value

NPV: negative predictive value

se.
